# Deadline-Aware Graph-Guided Beam Hopping and Precoding for Dynamic Low Earth Orbit Downlinks

**DOI:** 10.3390/s26216733

**Published:** 2026-10-24

**Authors:** Chenyu Wu, Yuhang Li, Baoji Wang, Guocheng Lv, Ye Jin

**Affiliations:** 1 School of Electronics, Peking University, Beijing 100871, China; wuchenyu@stu.pku.edu.cn (C.W.); yuhangli@stu.pku.edu.cn (Y.L.); lv.guocheng@pku.edu.cn (G.L.); jinye@pku.edu.cn (Y.J.); 2 National Computer Network Emergency Response Technical Team/Coordination Center of China, Beijing 100029, China

**Keywords:** low earth orbit (LEO) satellites, beam hopping, resource allocation, deadline-aware scheduling, interference graph, fractional programming

## Abstract

Dynamic multibeam low Earth orbit (LEO) downlinks must serve nonuniform traffic demand under power and spatial-multiplexing constraints. Beam hopping redistributes capacity across the satellite footprint, yet each cell-activation decision also reshapes co-channel interference. Aggregate backlog also conceals the remaining service opportunities of delay-sensitive traffic. We propose deadline-aware graph-guided fractional precoding (DAGFP), a causal cross-layer framework for joint cell activation and precoding. DAGFP maps age-resolved service slack into interference-compatible active-set construction, thereby linking queue urgency to the subchannel subsequently optimized by weighted fractional programming (FP). Precoding allocates power and suppresses residual interference under the same traffic weights. Under the normalized aperture model, DAGFP reduces the deadline-drop ratio by 5.51 percentage points relative to backlog-weighted Queue-Graph-FP at an offered-load factor of 2, starting from empty queues over a 1.024 s arrival window, illustrating the benefit of retaining traffic-age information under these conditions. Comparisons with independently tuned regularized zero-forcing (RZF) baselines also favor DAGFP at the evaluated congested loads. Controlled component comparisons further show that the contribution of age-resolved weighting depends on its placement in active-set construction and precoding. The benefit of age-resolved weighting depends on load, service overhead, and queue initialization: Queue-Graph-FP achieves lower drop at loads 8 and 36 in the main study and, in separate load-4 studies, under the tested overhead or warmed-up conditions. These findings indicate that coupling deadline urgency with channel-compatible active-set construction and subsequent precoding can improve timely service under suitable traffic and operating conditions in the evaluated LEO downlink model.

## 1. Introduction

Low Earth orbit (LEO) satellite networks are becoming an important component of non-terrestrial networks (NTNs). They extend broadband access beyond terrestrial coverage and support integrated space–air–ground services [1,2,3,4]. Multibeam high-throughput satellite payloads increase spatial reuse, while programmable regenerative payloads enable rapid reconfiguration of radio resources [5,6,7]. Beam hopping (BH) exploits this flexibility by changing the served-cell set over time. In this work, BH is implemented through binary cell-activation decisions. This operation is compatible with the Digital Video Broadcasting Satellite Second Generation Extension framework, and over-the-air experiments have demonstrated BH superframe synchronization [8,9]. Each activation decision, however, changes both the service opportunities of queued traffic and the co-channel interference among concurrent beams. Dynamic LEO downlink control must therefore coordinate traffic-aware scheduling with physical-layer precoding.

Satellite resource-allocation research has addressed coverage and frequency reuse [10], service-priority modeling, and metaheuristic beam scheduling [11,12]. Other studies balance throughput against the number of served users in frequency allocation [13] or develop online queue-aware resource control [14]. Conflict graphs and multidimensional decompositions reduce the combinatorial search over active cells [15,16,17]. Other formulations combine residual-demand scheduling with power allocation, use multiobjective optimization, or apply alternating procedures, including methods based on the alternating direction method of multipliers (ADMM), to BH design [18,19,20]. Earlier cross-layer studies coordinated packet scheduling with precoding [21] and combined BH with precoding under time-varying demand [22]. More recent work has addressed selective precoding [23], two-stage beam scheduling and power allocation [24], and alternating beam hopping and precoding optimization for dense LEO systems [25]. These methods demonstrate the value of coordinating discrete illumination with continuous radio-resource variables. These studies address demand matching, quality of service, energy, and aggregate system performance. Some also represent finite traffic lifetimes. The distinction examined here lies in how remaining service opportunities are incorporated into both channel-compatible selection and complex precoding.

Learning-based approaches use masked value methods, hybrid-action policy optimization, multi-agent coordination, and digital-twin-assisted control to learn hybrid resource-allocation policies [26,27,28,29]. Related designs combine deep reinforcement learning with unsupervised power models or geometric programming [30,31]. These approaches can represent complex state-to-action mappings, but they require training and validation over the intended operating conditions. Online optimization remains useful when queue priorities and interference relations must be directly interpretable.

These approaches motivate closer examination of the queue–channel interface when forming the active subchannel. Deadline-aware scheduling determines which traffic should be served, whereas interference-aware precoding determines how a selected set shares spatial resources. When these decisions are treated independently, activation may ignore channel compatibility, or scheduling priority may discard service slack. An urgent scheduler may then select strongly coupled cells, while a channel-driven optimizer may allocate rates efficiently without averting deadline violations. Discrete-time BH queuing models characterize throughput, occupancy, loss, and delay [32], but aggregate backlog cannot distinguish traffic with different remaining lifetimes. Cells with identical queued volumes may therefore require different actions. Expired traffic also leaves the queue even when it is not served, so bounded backlog alone does not imply timely delivery. Together, these observations motivate a tractable per-slot framework that carries deadline information into interference-compatible active-set construction before continuous precoder optimization.

To address this need, we develop deadline-aware graph-guided fractional precoding (DAGFP) for joint cell activation and precoding. Its central cross-layer mechanism transfers age-resolved service slack into interference-aware active-set construction rather than optimizing scheduling and precoding independently. The resulting active subchannel is then optimized by weighted fractional programming (FP) under the same traffic weights. A correlation graph and selected-subchannel precoder provide a tractable causal realization of this queue–channel interface under co-channel interference.

The study considers one satellite and a common deadline within each scenario; different deadlines are assessed in separate scenarios. Table 1 compares representative formulations already cited above.

The main contributions of this work are as follows.

- We formulate deadline-aware resource allocation by separating timely service from expiration loss and construct a causal cell-level urgency measure from the age-resolved queue. By retaining the remaining service slack that aggregate backlog discards, the formulation distinguishes backlog control from deadline reliability.
- DAGFP couples deadline urgency, instantaneous service opportunity, and pairwise channel compatibility when constructing the active set. The same urgency weights subsequently enter weighted precoding, allowing the queue state to influence both candidate selection and power allocation. A causal decomposition realizes this coupling through deterministic correlation screening followed by FP optimization over the reduced subchannel associated with the selected streams. It preserves stream-cardinality and total-power feasibility while restricting the iterative matrix factorizations to the selected-stream dimension.
- Controlled comparisons distinguish the roles of age information, channel-compatible selection and precoding. Five main baselines assess overall performance, while additional component controls identify when the coupling is beneficial. Under the reference conditions, the incremental benefit of age-resolved weighting over backlog weighting is associated primarily with active-set construction, alongside a distinct contribution from precoding. The aperture-model evaluation shows that these component effects depend on traffic and operating conditions, providing a basis for assessing where deadline-aware coupling is useful.

The remainder of this paper is organized as follows. Section 2 presents the LEO downlink, channel, traffic, and finite-lifetime queue models. Section 3 formulates the long-horizon objective and develops the DAGFP controller. Section 4 presents the reference evaluation and shared experimental framework. Section 5 evaluates performance, component roles, and sensitivity under the normalized aperture model, and Section 6 concludes the paper.

## 2. System Model

### 2.1. Network and Beam-Domain Representation

Two channel configurations share the queue model, decision variables, and aggregate port-power constraint. The parametric reference configuration independently specifies amplitude scales, angular coupling, and residual phases for the experiments in Section 4. The normalized aperture configuration links gain, beamwidth, and phase through an idealized feed geometry for the physical-performance comparisons in Section 5.

Consider the downlink of one multibeam LEO satellite over the slot set $T=\{0,\ldots ,t_{\max}-1\}$ and the cell set $N=\{0,\ldots ,n_{c}-1\}$. Each cell is represented by a single-antenna terminal. The normalized aperture configuration assumes $m=n_{c}$ separate circular apertures, each with one logical port and radio-frequency (RF) feed chain, indexed by M={0,…,m−1}. Aperture *p* tracks cell center *p*. Selecting a cell activates its associated feed and one data stream; the selected feeds jointly precode all selected streams. Inactive feeds transmit zero power.

All simultaneously served cells share an allocated downlink band of bandwidth $b_{\mathrm{sub}}$ under full frequency reuse. This assumption deliberately exposes co-channel interference and isolates the joint cell-activation and precoding problem from frequency partitioning. The payload can support at most $k_{\max}$ concurrent streams per slot.

Let $p_{\mathrm{sat},t}\in R^{3}$ and $p_{n}\in R^{3}$ denote the World Geodetic System 1984 (WGS-84) Earth-fixed Cartesian coordinates of the satellite and terminal *n* [33]. Their difference determines both the propagation distance and the off-axis geometry. In particular, the link vector and slant distance are

(1) $$d_{n,t}=p_{n}-p_{\mathrm{sat},t},\;d_{n,t}={∥d_{n,t}∥}_{2}.$$

Let $e_{n,t}$ denote the elevation angle from terminal *n* to the satellite and let $e_{\min}$ be the minimum service elevation. The binary visibility vector $m_{t}^{\mathrm{vis}}\in B^{n_{c}}$ is

(2) $${\left[m_{t}^{\mathrm{vis}}\right]}_{n}=I\left\{e_{n,t}\geq e_{\min}\right\}.$$

Visibility is an exogenous geometric state and restricts which cells may be activated.

Let $u_{n,t}=d_{n,t}/d_{n,t}$ and $\theta_{n,p,t}=\arccos\left(u_{n,t}^{⊤}u_{p,t}\right)$. We use the circular-aperture Bessel form discussed in 3rd Generation Partnership Project (3GPP) Technical Report (TR) 38.811 [1], with absolute gain fixed by a solid-angle normalization. For full half-power beamwidth (HPBW) $\theta_{\mathrm{HPBW}}$, define the signed field pattern

(3) $$f\left(\theta \right)=\left\{\begin{array}{cc} 2J_{1}\left(\kappa_{a}\sin\theta \right)/\left(\kappa_{a}\sin\theta \right),\; & 0<\theta \leq \pi /2,\\ 1, & \theta =0,\\ 0, & \pi /2<\theta \leq \pi , \end{array}\right.\;\kappa_{a}=\frac{u_{1/2}}{\sin(\theta_{\mathrm{HPBW}}/2)},$$

where $u_{1/2}$ is the first positive solution of ${\left[2J_{1}\left(u\right)/u\right]}^{2}=1/2$. Angles in trigonometric expressions are in radians. With radiation efficiency $\eta_{a}$, the field amplitude *F* and power gain *G* are

(4) $$\Omega_{a}=2\pi \int_{0}^{\pi /2}{\left|f\left(\theta \right)\right|}^{2}\sin\theta \;d\theta ,\;F\left(\theta \right)=\sqrt{\frac{4\pi \eta_{a}}{\Omega_{a}}}f\left(\theta \right),\;G\left(\theta \right)={\left|F\left(\theta \right)\right|}^{2}.$$

Thus $\int G\;d\Omega =4\pi \eta_{a}$; peak gain, beamwidth, and efficiency are not independent parameters. Sidelobe field signs are retained, with no imposed sidelobe floor. At $f_{c}=12$ GHz, $\theta_{\mathrm{HPBW}}=16{}^{\circ}$ and $\eta_{a}=0.6$, the diameter $D_{a}=\kappa_{a}\lambda_{c}/\pi$ is approximately 0.09236 m, and peak gain is 19.053 dBi, where $\lambda_{c}=299792458/f_{c}$ m. Transmit and receive apertures use these same parameters. Each receiver is modeled as a fixed-location ground terminal with a single directional receive aperture and ideal satellite tracking, so its receive off-axis angle is zero for every feed under the far-field common-direction approximation. Pointing errors and tracking dynamics are not modeled.

The 133 aperture centers occupy the first 133 positions of a row-major, 12-column planar grid, with spacing $2D_{a}$ and the grid centroid subtracted. Let $r_{p,t}$ be the displacement of aperture center *p* relative to $p_{\mathrm{sat},t}$, expressed in Earth-fixed axes. The local array normal points toward the Earth center; its first in-plane axis is the normalized cross product of the Earth rotation axis with that normal, and its second axis completes the right-handed basis. This geometry determines relative feed phases. The idealization excludes mutual coupling, matching networks, and amplifier dynamics. For a unit observation direction u, define the row field ${\left[f_{t}\left(u\right)\right]}_{p}=F\left(\arccos\left(u^{⊤}u_{p,t}\right)\right)\exp\left(j2\pi u^{⊤}r_{p,t}/\lambda_{c}\right)$ and the radiation matrix $R_{t}={\left(4\pi \right)}^{-1}\int_{S^{2}}f_{t}{\left(u\right)}^{H}f_{t}\left(u\right)\;d\Omega$. Its largest eigenvalue bounds radiated power per unit feed power, including coherent cross-feed terms. Sphere quadrature at four sampled orbital geometries gives maxima of 0.6202–0.6262, satisfying the necessary power bound at those geometries.

### 2.2. Full Complex Channel Model

The composite channel in slot *t* is represented by $H_{t}\in C^{n_{c}\times m}$. Its *n*th row follows the convention

(5) $${\left[H_{t}\right]}_{n,:}=h_{n,t}^{H},$$

where $h_{n,t}\in C^{m}$ is the column channel vector used in the subsequent signal model.

The large-scale linear channel power gain is

(6) $$h_{n,t}^{L}={\left(\frac{c_{0}}{4\pi f_{c}d_{n,t}}\right)}^{2}l_{n,t}^{\mathrm{rain}}l_{n,t}^{\mathrm{shadow}},$$

where $c_{0}=3\times 10^{8}$ m/s is the light-speed approximation used in the free-space link budget. A geographic region is one H3 resolution-2 parent containing seven resolution-3 child-cell centers. There are 19 such regions; preserving the child ordering gives region index r(n)=⌊n/7⌋. Rain and shadow have independent regional latent fields, with independent innovations across regions and common innovations within each region. For either stationary unit-normal latent field,

(7) $$x_{r,t}=\rho_{x}x_{r,t-1}+\sqrt{1-\rho_{x}^{2}}\varepsilon_{r,t},\;\rho_{x}=\exp\left(-\Delta t/\tau_{x}\right),\;\varepsilon_{r,t}∼N\left(0,1\right).$$

The rain and shadow time constants are 2 s and 0.5 s, respectively. Each latent process starts with an independent N(0,1) draw, so the first-order autoregressive (AR(1)) process starts in its stationary marginal distribution. Zenith rain loss in dB is $A_{r,t}^{z}=\exp\left(\mu_{A}+\sigma_{A}x_{r,t}^{\mathrm{rain}}\right)$, with mean $m_{A}=1$ dB, standard deviation $s_{A}=1$ dB, $\mu_{A}=\ln\left[m_{A}^{2}/\sqrt{m_{A}^{2}+s_{A}^{2}}\right]$, and $\sigma_{A}=\sqrt{\ln(1+s_{A}^{2}/m_{A}^{2})}$. The terminal-specific power multipliers are

(8) $$A_{n,t}=\mathrm{clip}\;\left(\frac{A_{r(n),t}^{z}}{\sin(\max\left(e_{n,t},1^{{}^{\circ}}\right))},0,20\right),\;l_{n,t}^{\mathrm{rain}}=10^{-A_{n,t}/10},\;l_{n,t}^{\mathrm{shadow}}=10^{-2x_{r(n),t}^{\mathrm{shadow}}/10}.$$

Shadow loss is unclipped, giving a linear multiplier with median one and mean approximately 1.11. Cells share their regional latent fields, although elevation scaling can give them different rain multipliers. Spatial dependence is therefore piecewise regional.

Rician models describe a line-of-sight (LoS) component plus diffuse scattering [1,4]. We assume $k_{n,t}^{\mathrm{ric}}=10^{[25+5e_{n,t}/90^{{}^{\circ}}]/10}$, an idealized strong-LoS setting. Each terminal has one complex fading process shared across feeds. Its zero-mean diffuse component is

(9) $$z_{n,t}^{\mathrm{ric}}=\rho_{\mathrm{ric}}z_{n,t-1}^{\mathrm{ric}}+\sqrt{1-\rho_{\mathrm{ric}}^{2}}\varepsilon_{n,t}^{\mathrm{ric}},\;\varepsilon_{n,t}^{\mathrm{ric}}∼\mathrm{CN}\left(0,1\right),$$

with $z_{n,0}^{\mathrm{ric}}∼\mathrm{CN}\left(0,1\right)$, independent terminal innovations, and $\rho_{\mathrm{ric}}=\exp\left(-\Delta t/\tau_{\mathrm{ric}}\right)$, $\tau_{\mathrm{ric}}=5$ ms.

After common carrier-phase and Doppler tracking, the residual phases $\psi_{n}$ are sampled independently across terminals from U[0,2π), independently of the diffuse initial states and innovations. Each $\psi_{n}$ remains fixed within a realization and is shared across all feeds for terminal *n*.

The shared factor is

(10) $$h_{n,t}^{S}=\sqrt{\frac{k_{n,t}^{\mathrm{ric}}}{k_{n,t}^{\mathrm{ric}}+1}}\exp\left(j\psi_{n}\right)+\sqrt{\frac{1}{k_{n,t}^{\mathrm{ric}}+1}}z_{n,t}^{\mathrm{ric}}.$$

Conditioned on the geometry and residual phase $\psi_{n}$, $h_{n,t}^{S}$ has unit mean power and LoS-to-diffuse power ratio $k_{n,t}^{\mathrm{ric}}$. The time constant $\tau_{\mathrm{ric}}=5$ ms describes the diffuse component. Port dependence enters through both aperture amplitudes and geometric phase,

(11) $${\left[H_{t}\right]}_{n,p}=F\left(0\right)F\left(\theta_{n,p,t}\right)\sqrt{h_{n,t}^{L}}\;h_{n,t}^{S}\exp\;\left(j\frac{2\pi }{\lambda_{c}}u_{n,t}^{⊤}r_{p,t}\right).$$

The positive phase sign follows the far-field distance $d_{n,p,t}≃d_{n,t}-u_{n,t}^{⊤}r_{p,t}$ after removing the common propagation phase. All propagation states are exogenous to resource allocation.

These parameters define a synthetic strong-LoS scenario with moderate amplitude variability. Both channel configurations use the same rain, shadowing, and Rician-factor parameters.

### 2.3. Parametric Reference Channel Configuration

The parametric reference assigns one cell-associated port per terminal and specifies its angular response independently of a physical feed layout. Recommendation S.1528 of the International Telecommunication Union Radiocommunication Sector (ITU-R) provides context for direction-dependent satellite antenna patterns [34]; the independent gain and width below are reference parameters. With the nominal angular-width parameter $\theta_{\mathrm{HPBW}}$ in degrees, set $d_{\mathrm{bw}}=58.44\lambda_{c}/\theta_{\mathrm{HPBW}}$, and define

(12) $$u=\pi \left(d_{\mathrm{bw}}/\lambda_{c}\right)\sin\theta ,\;G_{\mathrm{ref}}\left(\theta \right)=10^{g_{\max}/10}\max\left\{{\left[2J_{1}\left(u\right)/u\right]}^{2},10^{-a_{\mathrm{sl}}/10}\right\},$$

using the continuous limit at u=0. Both transmit and receive patterns use $g_{\max}=40$ dB and nominal angular width $\theta_{\mathrm{HPBW}}=16{}^{\circ}$, with suppression parameters $a_{\mathrm{sl}}=20$ dB and 25 dB, respectively. The receiver tracks the satellite. These independently specified quantities define a port-channel reference rather than a jointly realizable antenna gain and beamwidth.

In this reference, each terminal–port pair has an independently sampled residual phase $\psi_{n,p}∼U\left[0,2\pi \right)$, fixed within a realization, and its own diffuse process with independent complex-normal innovations. The large-scale factors and Rician coefficient follow the definitions above, but the pair-specific small-scale term and full channel are

(13) $$\begin{array}{cc} h_{n,p,t}^{S,ref} & =\sqrt{\frac{k_{n,t}^{\mathrm{ric}}}{k_{n,t}^{\mathrm{ric}}+1}}e^{j\psi_{n,p}}+\sqrt{\frac{1}{k_{n,t}^{\mathrm{ric}}+1}}z_{n,p,t}^{\mathrm{ric}},\\ {}[H_{t}^{\mathrm{ref}}{]}_{n,p} & =\sqrt{G_{\mathrm{ref}}^{\mathrm{rx}}\left(0\right)G_{\mathrm{ref}}^{\mathrm{tx}}\left(\theta_{n,p,t}\right)h_{n,t}^{L}}\;h_{n,p,t}^{S,ref}. \end{array}$$

### 2.4. Signal, Rate, and Power Model

Cell activation is represented by $\alpha_{n,t}\in B=\left\{0,1\right\}$, with $\alpha_{n,t}=1$ if cell *n* is scheduled at slot *t*. Consequently, the payload stream budget imposes

(14) $$\sum_{n=0}^{n_{c}-1}\alpha_{n,t}\leq k_{\max}.$$

Let $w_{n,t}\in C^{m}$ be the precoder for the normalized data symbol $\xi_{n,t}$, where $E[|\xi_{n,t}{|}^{2}]=1$. The data symbols are zero-mean and mutually uncorrelated across streams. The inactive columns are fixed to 0, and $W_{t}=\left[w_{0,t},\ldots ,w_{n_{c}-1,t}\right]\in C^{m\times n_{c}}$. The transmitted port-domain signal is

(15) $$x_{t}=\sum_{n=0}^{n_{c}-1}\alpha_{n,t}w_{n,t}\xi_{n,t}.$$

The received baseband signal at cell *n* is

(16) $$y_{n,t}=\alpha_{n,t}h_{n,t}^{H}w_{n,t}\xi_{n,t}+\sum_{k\in N\backslash \{n\}}\alpha_{k,t}h_{n,t}^{H}w_{k,t}\xi_{k,t}+z_{n,t}^{\mathrm{rx}},$$

where $z_{n,t}^{\mathrm{rx}}∼\mathrm{CN}\left(0,\sigma^{2}\right)$ and

(17) $$\sigma^{2}=10^{N_{0}/10}b_{\mathrm{sub}}.$$

Here, $N_{0}$ is the effective noise power spectral density in dBW/Hz, which includes the receiver-noise assumption of the link model. Treating co-channel interference as noise, the signal-to-interference-plus-noise ratio (SINR) and achievable rate are

(18) $$\begin{array}{cc} \gamma_{n,t} & =\frac{\alpha_{n,t}{\left|h_{n,t}^{H}w_{n,t}\right|}^{2}}{\sum_{k\in N\backslash \{n\}}\alpha_{k,t}{\left|h_{n,t}^{H}w_{k,t}\right|}^{2}+\sigma^{2}}, \end{array}$$

(19) $$\begin{array}{cc} r_{n,t} & =\frac{b_{\mathrm{sub}}}{10^{6}}\log_{2}\left(1+\gamma_{n,t}\right)\;\mathrm{Mbps}. \end{array}$$

The service capacity and transmit power in one slot are

(20) $$c_{n,t}=r_{n,t}\Delta t\;\mathrm{Mb},\;p_{t}=\sum_{n=0}^{n_{c}-1}{∥w_{n,t}∥}_{2}^{2}.$$

In the parametric reference configuration, $p_{t}$ is the reference port power. In the normalized aperture configuration, the same quadratic form is aggregate feed RF output. It is neither angle-integrated radiated power nor electrical consumption. Let $E_{K}$ contain the coordinate vectors of the selected feeds and let $V\in C^{k_{t}\times k_{t}}$ be their digital precoder. The full action is $W_{t}=E_{K}VE_{K}^{⊤}$. Since $E_{K}^{⊤}E_{K}=I$, independent unit-power symbols give $E{∥x_{t}∥}_{2}^{2}={∥W_{t}∥}_{F}^{2}={∥V∥}_{F}^{2}$. Feed *p* carries $\sum_{n}{\left|{\left[W_{t}\right]}_{p,n}\right|}^{2}$, whereas stream *n* carries the squared norm of the corresponding column. The main model constrains their sum to 1000 W without a separate tighter per-amplifier rating. The selected-feed and stream counts coincide in this architecture, while their common cardinality limit and the aggregate RF-power budget constrain different resources.

### 2.5. Deadline-Constrained Traffic and Queue Dynamics

All traffic within a scenario has the same lifetime $d_{\max}\Delta t$. Different lifetimes are evaluated in separate scenarios. Let $\lambda_{\min}^{\left(0\right)}$ and $\lambda_{\max}^{\left(0\right)}$ denote the lower and upper mean-rate bounds at unit offered load. For load factor χ, define $\lambda_{\min}\left(\chi \right)=\chi \lambda_{\min}^{\left(0\right)}$ and $\lambda_{\max}\left(\chi \right)=\chi \lambda_{\max}^{\left(0\right)}$. In each stationary realization, the mean offered rate of cell *n* is independently sampled as $\lambda_{n}∼U\left[\lambda_{\min}\left(\chi \right),\lambda_{\max}\left(\chi \right)\right]$ and remains fixed throughout the realization. Conditional on $\lambda_{n}$, the bit-level arrival volume is generated as

(21) $$x_{n,t}^{\mathrm{arr}}∼\mathrm{Poisson}\left(\lambda_{n}10^{6}\Delta t\right),\;a_{n,t}=10^{-6}x_{n,t}^{\mathrm{arr}}\;\mathrm{Mb}.$$

This model represents aggregate traffic volume and does not require a separate packet-size assumption. Collecting the per-cell arrivals gives the column vector $a_{t}={\left[a_{0,t},\ldots ,a_{n_{c}-1,t}\right]}^{⊤}$.

Let $d_{\max}\geq 2$ be the number of age stages. The matrix $Q_{t}\in R_{+}^{n_{c}\times d_{\max}}$ denotes the queue state immediately before resource allocation in slot *t*. Element ${\left[Q_{t}\right]}_{n,d}$ is the traffic volume in Mb that has waited for $d\in \{0,\ldots ,d_{\max}-1\}$ slots. The initial arrival $a_{n,0}$ occupies age stage zero before the first allocation. Service is then applied in decreasing order of age. With $S_{t}\in R_{+}^{n_{c}\times d_{\max}}$, the served amount is

(22) $${\left[S_{t}\right]}_{n,d}=\min\left({\left[Q_{t}\right]}_{n,d},\max\left(0,c_{n,t}-\sum_{j=d+1}^{d_{\max}-1}{\left[S_{t}\right]}_{n,j}\right)\right).$$

After service, the residual traffic in the oldest stage expires:

(23) $$l_{n,t}={\left[Q_{t}\right]}_{n,d_{\max}-1}-{\left[S_{t}\right]}_{n,d_{\max}-1}.$$

The remaining traffic ages, and the arrival of slot t+1 is inserted before the next action:

(24) $$\begin{array}{cccc} {\left[Q_{t+1}\right]}_{n,d} & ={\left[Q_{t}\right]}_{n,d-1}-{\left[S_{t}\right]}_{n,d-1},\; & \; & d\in \{1,\ldots ,d_{\max}-1\}, \end{array}$$

(25) $$\begin{array}{cc} {}[Q_{t+1}{]}_{n,0} & =a_{n,t+1}. \end{array}$$

The aggregate backlog and timely service of cell *n* are obtained by summing over the age stages:

(26) $$q_{n,t}=\sum_{d=0}^{d_{\max}-1}{\left[Q_{t}\right]}_{n,d},\;s_{n,t}=\sum_{d=0}^{d_{\max}-1}{\left[S_{t}\right]}_{n,d}.$$

For every nonterminal transition, the exact conservation identity is

(27) $$q_{n,t+1}=q_{n,t}-s_{n,t}-l_{n,t}+a_{n,t+1}.$$

Expired traffic is excluded from useful service. Finite-horizon evaluation appends a deadline-length guard interval so that every offered arrival receives its complete service and expiration horizon, as detailed in Section 5.1.

## 3. Deadline-Aware Graph-Guided Fractional Precoding

### 3.1. Long-Term Design Objective

Because expiration removes old traffic, a finite-lifetime queue need not exhibit secular accumulation even when deadline loss is high. Queue stability is therefore not used as a reliability objective. Instead, the system-level design is expressed through the long-term average drop volume and transmit power,

(28) $$\begin{array}{cc} \bar{l} & =\underset{t_{\max}\to \infty }{\mathrm{lim sup}}\frac{1}{t_{\max}}\sum_{t=0}^{t_{\max}-1}E\left[\sum_{n=0}^{n_{c}-1}l_{n,t}\right], \end{array}$$

(29) $$\begin{array}{cc} \bar{p} & =\underset{t_{\max}\to \infty }{\mathrm{lim sup}}\frac{1}{t_{\max}}\sum_{t=0}^{t_{\max}-1}E\left[p_{t}\right]. \end{array}$$

At slot *t*, the controller has the causal information

(30) $$I_{t}=\left\{\left(Q_{\tau },H_{\tau },m_{\tau }^{\mathrm{vis}}\right):0\leq \tau \leq t\right\}.$$

The current arrival is already represented in $Q_{t}$. A causal policy $\pi ={\left\{\pi_{t}\right\}}_{t\geq 0}$ maps this information to $\left(\alpha_{t},W_{t}\right)=\pi_{t}\left(I_{t}\right)$. Here, causality means only that no future queue, channel, or visibility state enters the decision. Let C denote the class of policies whose induced actions satisfy the per-slot constraints below. For a fixed load factor χ, the reference volume $l_{\mathrm{ref}}=n_{c}\lambda_{\max}\left(\chi \right)\Delta t$ has units of Mb per slot and makes the reliability term dimensionless. Normalizing power by $p_{\max}$ gives the design objective

(31) $$\begin{array}{cc} P_{1}:\min_{\pi \in C}\; & \frac{\bar{l}}{l_{\mathrm{ref}}}+v\frac{\bar{p}}{p_{\max}}, \end{array}$$

(32) $$\begin{array}{cc} s.t.\; & \sum_{n=0}^{n_{c}-1}\alpha_{n,t}\leq k_{\max},\;\forall t, \end{array}$$

(33) $$\begin{array}{cc} \; & \alpha_{n,t}\in B,\;\forall n,t, \end{array}$$

(34) $$\begin{array}{cc} \; & \alpha_{n,t}\leq {\left[m_{t}^{\mathrm{vis}}\right]}_{n},\;\forall n,t, \end{array}$$

(35) $$\begin{array}{cc} \; & \sum_{n=0}^{n_{c}-1}{∥w_{n,t}∥}_{2}^{2}\leq p_{\max},\;\forall t, \end{array}$$

(36) $$\begin{array}{cc} \; & {∥w_{n,t}∥}_{2}^{2}\leq \alpha_{n,t}p_{\max},\;\forall n,t, \end{array}$$

(37) $$\begin{array}{cc} \; & |{\left[w_{n,t}\right]}_{p}{|}^{2}\leq \alpha_{p,t}p_{\max},\;\forall p,n,t. \end{array}$$

Constraints (32)–(33) impose the concurrent-stream limit and binary activation, while (34) excludes geometrically unavailable cells. Constraint (35) bounds the aggregate transmit power. The remaining two constraints couple the support of the precoder to the activation vector. Specifically, (36) sets column *n* of $W_{t}$ to zero when stream *n* is inactive. Because $m=n_{c}$ and port *p* is associated with cell *p*, (37) likewise sets row *p* to zero when that port is inactive. When the corresponding activation variable is one, these support constraints introduce no additional per-stream or per-element power budget. Constraint (35) already bounds each squared column norm and each squared entry magnitude by $p_{\max}$. Thus, (36) and (37) act as valid on–off support constraints and match the selected-row-and-column embedding used by the controller.

Here, v>0 is a dimensionless power weight. Problem $P_{1}$ specifies the intended reliability–power trade-off but is not optimized directly. DAGFP instead uses the following causal single-slot surrogate.

### 3.2. Age-Aware Single-Slot Surrogate

Let $q_{\mathrm{ref}}=\lambda_{\max}\left(\chi \right)d_{\max}\Delta t$ be the reference backlog in Mb. The normalized urgency is

(38) $$\omega_{n,t}=\frac{1}{q_{\mathrm{ref}}}\sum_{d=0}^{d_{\max}-1}\left(1+\beta \frac{d}{d_{\max}-d}\right){\left[Q_{t}\right]}_{n,d},$$

where β≥0 controls the additional priority assigned to older traffic. At age *d*, the denominator $d_{\max}-d$ is the number of service opportunities remaining including the current slot. The ratio $d/(d_{\max}-d)$ is zero for newly arrived traffic and grows increasingly rapidly as expiration approaches. It therefore acts as a deterministic proxy for deadline risk rather than an estimated expiration probability. The weight remains finite because $d\leq d_{\max}-1$. Empty cells have $\omega_{n,t}=0$, whereas β=0 yields a backlog-only weight. The slot-level surrogate is

(39) $$\begin{array}{cc} P_{2}:\max_{\alpha_{t},W_{t}}\;\; & \sum_{n=0}^{n_{c}-1}\alpha_{n,t}\omega_{n,t}\log_{2}\left(1+\gamma_{n,t}\right)-\frac{v}{p_{\max}}\sum_{n=0}^{n_{c}-1}{∥w_{n,t}∥}_{2}^{2},\\ s.t.\;\; & (32),\;(33),\;(34),\;(35),\;(36),\;(37). \end{array}$$

The reciprocal-slack term admits a limited service-demand interpretation. For one isolated cell, consider only currently queued divisible traffic, no future arrivals, and constant available service per slot. Write $D=d_{\max}$, $x_{r}={\left[Q_{t}\right]}_{n,D-r}$ and $F_{k}=\sum_{r=1}^{k}x_{r}$. The minimum constant service that can clear all current deadlines is $b_{*}=\max_{1\leq k\leq D}F_{k}/k$. Define $s_{\mathrm{clr}}=\sum_{r=1}^{D}x_{r}/r$ and the harmonic number $H_{D}=\sum_{r=1}^{D}1/r$. Then

(40) $$b_{*}\leq s_{\mathrm{clr}}\leq H_{D}b_{*},\;q_{\mathrm{ref}}\omega_{n,t}=\left(1-\beta \right)q_{n,t}+\beta Ds_{\mathrm{clr}}.$$

Indeed, deadline feasibility requires and, under oldest-first service, is ensured by $F_{k}\leq kb$ for every *k*. The lower bound follows from $s_{\mathrm{clr}}\geq F_{k}/k$; summation by parts gives $s_{\mathrm{clr}}=F_{D}/D+\sum_{k=1}^{D-1}F_{k}/\left[k\left(k+1\right)\right]\leq H_{D}b_{*}$. At β=1, urgency is therefore a normalized conservative clearance-demand indicator. For β>1 the original weights remain nonnegative, but the displayed identity is not a convex combination.

In the coupled wireless system, $P_{2}$ remains a surrogate for $P_{1}$: it uses current information and spectral efficiency in place of future queue consequences and capped backlog service.

### 3.3. Deadline-Aware Interference-Compatible Active-Set Construction

The first decision stage uses a deterministic heuristic graph screen to convert queue urgency and instantaneous channel compatibility into a tractable cell-activation rule. Exhaustive subset search can require evaluating up to ∑r=0kmaxncr candidates. For i,j∈N, define the normalized complex-channel correlation as

(41) $$c_{i,j,t}=\left\{\begin{array}{cc} \frac{|h_{i,t}^{H}h_{j,t}|}{∥h_{i,t}{∥}_{2}{∥h_{j,t}∥}_{2}},\; & i\neq j,\;∥{\bar{h}}_{i,t}{∥}_{2}{∥{\bar{h}}_{j,t}∥}_{2}>\epsilon_{n},\\ 0, & \mathrm{otherwise}. \end{array}\right.$$

In the validation implementation, before numerical selection or precoding, all methods use ${\bar{H}}_{t}=H_{t}/\sigma$ and unit noise variance. This equivalent receiver-noise normalization leaves SINR unchanged and makes the numerical floor $\epsilon_{n}=10^{-12}$ apply to normalized, rather than physical, channel norms. The numerator and denominator of (41) can equivalently use normalized rows. The graph $G_{t}=\left(N,E_{t}\right)$ contains edge (i,j) when $c_{i,j,t}>\eta$. Adjacent vertices are excluded from simultaneous selection. This threshold is a heuristic compatibility rule, not a physical infeasibility condition.

The channel-quality proxy and combined score are

(42) $${\hat{u}}_{n,t}=\log_{2}\left(1+\frac{p_{\max}{∥h_{n,t}∥}_{2}^{2}}{k_{\max}\sigma^{2}}\right),\;\zeta_{n,t}=\omega_{n,t}{\hat{u}}_{n,t}.$$

The equal-power term $p_{\max}/k_{\max}$ is used only for ranking. The product $\omega_{n,t}{\hat{u}}_{n,t}$ is a separable proxy for urgency-weighted service opportunity before multiuser interference is known. Correlation screening then removes strongly coupled candidates, and the continuous stage resolves the residual interference among the selected cells. Invisible, empty, or numerically zero-channel cells are excluded. Ties are broken by the smaller cell index.

If the graph admits fewer than $k_{\max}$ eligible mutually compatible cells, the smaller returned set remains feasible. Computing $H_{t}H_{t}^{H}$ costs $O(n_{c}^{2}m)$, and deterministic score ordering with direct compatibility checks costs $O(n_{c}\log n_{c}+k_{\max}n_{c})$. This greedy compatibility heuristic is evaluated against exhaustive subset enumeration on reduced states in Section 5.3.

### 3.4. Weighted Fractional-Programming Precoding

Graph selection determines which cells can share a slot, but it does not allocate power or suppress the residual interference within that set. The second stage therefore optimizes the precoder on the selected subchannel. Let $k_{t}=\left|K_{t}\right|$ and order $K_{t}=\left\{n_{0},\ldots ,n_{k_{t}-1}\right\}$. Because cells and transmit ports have a one-to-one index correspondence, the continuous stage uses the principal submatrix

(43) $${\tilde{H}}_{t}={\left[H_{t}\right]}_{K_{t},K_{t}}\in C^{k_{t}\times k_{t}},$$

where the row associated with selected cell *n* is denoted by ${\tilde{h}}_{n,t}^{H}$. Because the active set is not yet known, the graph-stage correlation in (41) uses complete candidate-port rows as a preselection proxy for potential coupling. After $K_{t}$ is fixed, inactive ports are zeroed and the continuous stage uses the corresponding principal subchannel. The two channel objects therefore serve distinct decision stages. Let ${\tilde{w}}_{n,t}\in C^{k_{t}}$ be the reduced precoder of selected stream *n*. Conditioned on $K_{t}$, the continuous optimization stage balances weighted spectral efficiency against a dimensionless normalized power penalty through

(44) $$\begin{array}{cc} P_{3}:\max_{{\left\{{\tilde{w}}_{n,t}\right\}}_{n\in K_{t}}}\;\; & \sum_{n\in K_{t}}\omega_{n,t}\log_{2}\left(1+\gamma_{n,t}\right)-\frac{v}{p_{\max}}\sum_{n\in K_{t}}{∥{\tilde{w}}_{n,t}∥}_{2}^{2}, \end{array}$$

(45) $$\begin{array}{cc} s.t.\; & \sum_{n\in K_{t}}{∥{\tilde{w}}_{n,t}∥}_{2}^{2}\leq p_{\max}. \end{array}$$

To separate the desired signal from the interference-plus-noise term, introduce

(46) $$a_{n,t}^{\mathrm{sig}}=|{\tilde{h}}_{n,t}^{H}{\tilde{w}}_{n,t}{|}^{2},\;i_{n,t}^{\mathrm{in}}=\sum_{k\in K_{t}\backslash \left\{n\right\}}{\left|{\tilde{h}}_{n,t}^{H}{\tilde{w}}_{k,t}\right|}^{2}+\sigma^{2},\;d_{n,t}^{\mathrm{tot}}=a_{n,t}^{\mathrm{sig}}+i_{n,t}^{\mathrm{in}},$$

together with $\kappa_{n,t}=\omega_{n,t}/\ln2$. The following reformulation applies the Lagrangian dual and quadratic transforms in [35]. Introduce the nonnegative auxiliary variable $\mu_{n,t}$. The logarithmic dual transform gives

(47) $$\begin{array}{cc} f_{a}\left({\tilde{W}}_{t},\mu_{t}\right)= & \sum_{n\in K_{t}}\kappa_{n,t}\left[\ln\left(1+\mu_{n,t}\right)-\mu_{n,t}+\left(1+\mu_{n,t}\right)\frac{a_{n,t}^{\mathrm{sig}}}{d_{n,t}^{\mathrm{tot}}}\right]\\ \; & -\frac{v}{p_{\max}}\sum_{n\in K_{t}}{∥{\tilde{w}}_{n,t}∥}_{2}^{2}. \end{array}$$

For a fixed precoder, maximizing (47) over $\mu_{n,t}$ yields

(48) $$\mu_{n,t}=\frac{a_{n,t}^{\mathrm{sig}}}{i_{n,t}^{\mathrm{in}}}=\gamma_{n,t}.$$

The remaining signal-to-total-power ratio is then decoupled by the complex quadratic transform, giving

(49) fb(W˜t,μt,φt)=∑n∈Ktκn,t[ln(1+μn,t)−μn,t]+ 2∑n∈KtReφn,t*κn,t(1+μn,t)h˜n,tHw˜n,t−∑n∈Kt|φn,t|2dn,ttot−vpmax∑n∈Kt∥w˜n,t∥22.

For fixed ${\tilde{W}}_{t}$ and $\mu_{t}$, the corresponding complex auxiliary maximizer is

(50) $$\varphi_{n,t}=\frac{\sqrt{\kappa_{n,t}\left(1+\mu_{n,t}\right)}{\tilde{h}}_{n,t}^{H}{\tilde{w}}_{n,t}}{d_{n,t}^{\mathrm{tot}}}.$$

Finally, with ν≥0 denoting the Lagrange multiplier of the total-power constraint, the stationary maximizer of the concave precoder block is

(51) $$\begin{array}{cc} {\tilde{w}}_{n,t}\left(\nu \right)= & {\left(\sum_{k\in K_{t}}{\left|\varphi_{k,t}\right|}^{2}{\tilde{h}}_{k,t}{\tilde{h}}_{k,t}^{H}+\left(\frac{v}{p_{\max}}+\nu \right)I_{k_{t}}\right)}^{-1}\\ \; & \times \sqrt{\kappa_{n,t}\left(1+\mu_{n,t}\right)}\varphi_{n,t}{\tilde{h}}_{n,t}. \end{array}$$

Let $p\left(\nu \right)=\sum_{n\in K_{t}}{∥{\tilde{w}}_{n,t}\left(\nu \right)∥}_{2}^{2}$. Since v>0, the matrix in (51) is positive definite; p(ν) is continuous, nonincreasing, and tends to zero. We use ν=0 if feasible; otherwise, we double an upper bracket and bisect toward a feasible multiplier. Both loops allow at most 80 iterations. A fixed absolute multiplier-width tolerance can be misleading when channel scales change, so the solver instead checks feasibility and an objective-based optimality bound.

For fixed auxiliary variables, let $W\in C^{k_{t}\times k_{t}}$ denote the reduced precoder and use the matrix inner product $\langle X,Y\rangle =\mathrm{Tr}\left(X^{H}Y\right)$. Write the concave block as Q(W)=2Re〈B,W〉−〈W,AW〉−c∥W∥F2, where $c=v/p_{\max}>0$, $A=\sum_{k\in K_{t}}{\left|\varphi_{k,t}\right|}^{2}{\tilde{h}}_{k,t}{\tilde{h}}_{k,t}^{H}⪰0$, and the column of B associated with stream *n* is $\sqrt{\kappa_{n,t}\left(1+\mu_{n,t}\right)}\varphi_{n,t}{\tilde{h}}_{n,t}$. Let $Q^{*}$ be the maximum of *Q* subject to ${∥W∥}_{F}^{2}\leq p_{\max}$. For feasible W and ν≥0,

(52) $$Q^{*}-Q\left(W\right)\leq \nu (p_{\max}-{∥W∥}_{F}^{2})+\frac{{∥B-\left[A+\left(c+\nu \right)I\right]W∥}_{F}^{2}}{\lambda_{\min}\left(A\right)+c+\nu }.$$

This follows by completing the square in the Lagrangian and using ${\left[A+\left(c+\nu \right)I\right]}^{-1}⪯{\left[\lambda_{\min}\left(A\right)+c+\nu \right]}^{-1}I$. The solver requires the right-hand side divided by max(1,|Q(W)|) to be at most $10^{-10}$; failure to reach this condition is reported rather than accepted as convergence. It concerns only the fixed-auxiliary concave block, not the global optimality of FP or joint selection.

One Hermitian eigendecomposition serves all multiplier trials. Caching squared projected right-hand-side row norms reduces each trial power evaluation to a sum over $k_{t}$ eigenvalues. Candidate reconstruction and the residual check in (52) occur when the spectral complementarity term is sufficiently small. All FP comparators in the validation studies use the same accuracy criterion. Section 4.2 specifies the settings used in the reference experiments.

Using the noise-normalized selected channel ${\bar{\tilde{H}}}_{t}={\tilde{H}}_{t}/\sigma$, the regularized zero-forcing (RZF) initialization forms

(53) $$W_{\mathrm{rzf},t}={\bar{\tilde{H}}}_{t}^{H}{\left({\bar{\tilde{H}}}_{t}{\bar{\tilde{H}}}_{t}^{H}+\delta_{\mathrm{rzf}}I_{k_{t}}\right)}^{-1}.$$

If ${∥W_{\mathrm{rzf},t}∥}_{F}^{2}\leq \epsilon_{n}$, the implementation uses ${\bar{\tilde{H}}}_{t}^{H}$ as the base matrix. Otherwise, it uses $W_{\mathrm{rzf},t}$. Denoting this nondegenerate base by $W_{\mathrm{base},t}$, the power-normalized initialization is

(54) $${\tilde{W}}_{t}^{\left(0\right)}=\frac{\sqrt{p_{\max}}}{∥W_{\mathrm{base},t}{∥}_{F}}W_{\mathrm{base},t}.$$

If the fallback also has squared Frobenius norm no greater than $\epsilon_{n}$, the implementation terminates the update with a numerical error. The complete rule is $\delta_{\mathrm{rzf}}\left(\rho \right)=\max\left\{\rho \mathrm{Tr}\left({\bar{\tilde{H}}}_{t}{\bar{\tilde{H}}}_{t}^{H}\right)/k_{t},10^{-12}\right\}$. FP initialization uses $\rho =10^{-3}$; baseline coefficients and ablation settings are specified in Section 5.1. The reference implementation uses the same trace rule on unnormalized channels. When absolute safeguards are inactive, positive channel scaling scales the Gram matrix and regularizer together, and total-power normalization cancels the resulting inverse scale of the RZF base. The absolute floor can break this invariance; changes in physical signal-to-noise ratio (SNR) and spatial structure can separately change the best relative coefficient. After optimization, the reduced rows and columns are embedded at indices $K_{t}$ of the full $m\times n_{c}$ matrix $W_{t}$, while all other entries remain zero. Let $f_{t}^{\left(i\right)}$ be the original objective (44). The stopping residual is $|f_{t}^{\left(i\right)}-f_{t}^{\left(i-1\right)}\left|/\max(1,\right|f_{t}^{\left(i-1\right)}\left|\right)$.

Under exact block maximization, the transformed formulation has the standard block-ascent property. Because finite multiplier bisection and floating-point arithmetic introduce numerical error, the implementation evaluates the original objective after every candidate update and accepts only iterates that are nondecreasing within tolerance. Section 5.7 reports stationarity, stopping, and computational diagnostics.

Computing urgency costs $O(n_{c}d_{\max})$; the complete Gram matrix costs $O(n_{c}^{2}m)$; score sorting and compatibility checks cost $O(n_{c}\log n_{c}+k_{\max}n_{c})$. For $k_{t}$ selected cells, RZF, the common FP matrix, and its eigendecomposition each cost $O(k_{t}^{3})$. Cached multiplier power evaluations cost $O(k_{t})$ each, whereas dense candidate reconstruction and accuracy checks cost $O(k_{t}^{3})$. Let $i_{\mathrm{fp}}\leq i_{\max}$ denote the number of executed FP updates, and let $i_{b}$ and $i_{c}$ bound all power evaluations and dense accuracy checks per update, respectively. The decision cost is

(55) $$O\;\left(n_{c}d_{\max}+n_{c}^{2}m+n_{c}\log n_{c}+k_{\max}n_{c}+\left[1+i_{\mathrm{fp}}\left(1+i_{c}\right)\right]k_{t}^{3}+i_{\mathrm{fp}}i_{b}k_{t}+mn_{c}\right).$$

The dense $m\times n_{c}$ output contributes $O(mn_{c})$. Auxiliary memory is $O(n_{c}d_{\max}+n_{c}^{2}+k_{t}^{2}+mn_{c})$, excluding input states and persistent histories. The stopping rule returns the last accepted feasible solution. Its stationarity, service performance and execution time are assessed separately in Section 5.7, since a finite iteration budget alone guarantees neither stationarity nor a wall-clock deadline.

### 3.5. End-to-End Causal Decision Procedure

**Figure 1:**
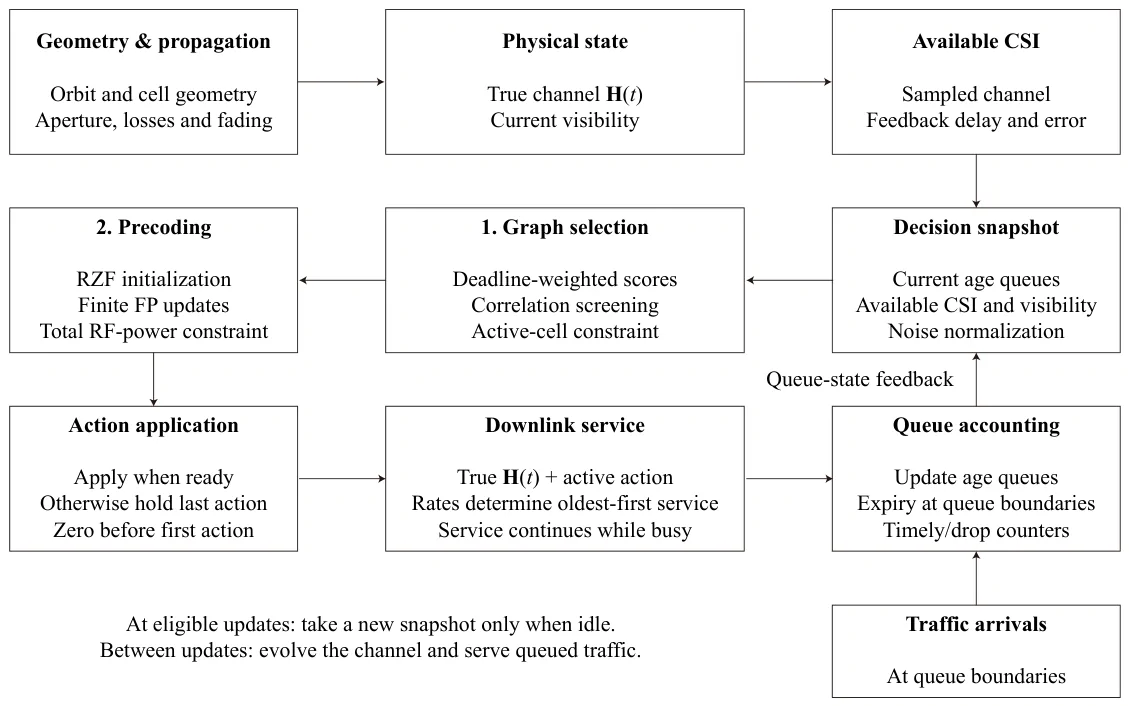
Causal state–decision–service flow. Geometry, propagation, and traffic are exogenous. The controller uses the observed CSI and current queue; graph selection and FP produce an action that is applied only when available. Actual service uses the true channel, and oldest-first service, expiry, and aging determine the next queue. The timing layer separates feedback, computation, and action application from the environment’s integration step. Abbreviations: CSI, channel state information; RZF, regularized zero-forcing; FP, fractional programming; RF, radio frequency.

Figure 1 connects the state, decision, and service models. Algorithm 1 describes their ideal instantaneous-computation chronology. The current arrival is present when resource allocation is computed. Service precedes the expiration, queue aging, and insertion of the next arrival. Delayed computation changes action application as specified below, not the queue’s lifetime convention.
**Algorithm 1:** End-to-End DAGFP decision procedure**Input:** η, β, *v*, $k_{\max}$, $d_{\max}$, $p_{\max}$, and $t_{\max}$. **Initialize:** Insert $a_{0}$ into age stage 0 of $Q_{0}$ and set t←0.
  1:**while** $t<t_{\max}$ **do**  2:    Observe $H_{t}$ and the visibility vector.  3:    Compute $q_{t}$ and $\omega_{t}$ using (26) and (38).  4:    Construct $G_{t}$ and $\zeta_{t}$ using (41) and (42).  5:    Invoke Algorithm 2 to obtain $K_{t}$ and $\alpha_{t}$.  6:    **if** $K_{t}=\emptyset$ **then**  7:        Set $W_{t}\leftarrow 0$ and $\gamma_{n,t}\leftarrow 0$, $r_{n,t}\leftarrow 0$, $c_{n,t}\leftarrow 0$ for every cell.  8:    **else**  9:       Invoke Algorithm 3 and embed its selected rows and columns into the full $m\times n_{c}$ precoding matrix.10:        Evaluate $\gamma_{n,t}$, $r_{n,t}$, and $c_{n,t}$ using (18)–(20).11:    **end if**12:    Apply oldest-first service and compute $s_{n,t}$ and $l_{n,t}$.13:    If $t+1<t_{\max}$, age residual traffic and insert $a_{t+1}$ using (24) and (25).14:    Record service, drops, power, and active cells. Set t←t+1.15:**end while**


The applied action is cast to the simulator’s complex64 transmit format. If quantization raises the transmit power above the aggregate budget, a common radial backoff restores feasibility; measured action-construction cost and reported RF power include this step. Selection and optimization otherwise use complex128 arithmetic.
**Algorithm 2:** Deterministic DAGFP cell selection**Input:** $G_{t}$, $\zeta_{t}$, $q_{t}$, $H_{t}$, visibility vector $m_{t}^{\mathrm{vis}}$, $k_{\max}$, and numerical floor $\epsilon_{n}$. **Output:** Selected set $K_{t}$ and map $\alpha_{t}$.
**Initialize:** $U\leftarrow \{n\in N|q_{n,t}>\epsilon_{n},\;{\left[m_{t}^{\mathrm{vis}}\right]}_{n}=1,\;∥{\bar{h}}_{n,t}{∥}_{2}>\epsilon_{n}\}$, $K_{t}\leftarrow \emptyset$, $\alpha_{t}\leftarrow 0$.
 1:**while** $|K_{t}|\;<k_{\max}$ **and** U≠∅ **do** 2:   $n^{★}\leftarrow \arg\max_{n\in U}\zeta_{n,t}$. 3:   Resolve ties by the smallest cell index. 4:   $K_{t}\leftarrow K_{t}\cup \left\{n^{★}\right\}$ and $\alpha_{n^{★},t}\leftarrow 1$. 5:   $U\leftarrow U\backslash \left(\left\{n^{★}\right\}\cup \left\{j|\left(n^{★},j\right)\in E_{t}\right\}\right)$. 6:**end while** 7:Return $K_{t}$ and $\alpha_{t}$.


**Algorithm 3:** Weighted FP precoding in DAGFP**Input:** $K_{t}$, $\omega_{t}$, $H_{t}$, *v*, $p_{\max}$, $i_{\max}$, $i_{b,max}$, $\epsilon_{\mathrm{fp}}$, the inner gap target, and $\epsilon_{\mathrm{mon}}$. **Output:** Feasible ${\tilde{W}}_{t}$ and embedded $W_{t}$.
**Initialize:** ${\tilde{W}}_{t}^{\left(0\right)}$ by (53) and (54), evaluate $f_{t}^{\left(0\right)}$, and set i←0.
  1:**repeat**  2:    Update every $\mu_{n,t}$ by (48).  3:    Update every $\varphi_{n,t}$ by (50).  4:    Evaluate p(0) using (51).  5:    **if** $p\left(0\right)\leq p_{\max}$ **then**  6:       Set $\nu^{★}\leftarrow 0$.  7:    **else**  8:       Set $\nu_{\mathrm{lo}}\leftarrow 0$ and $\nu_{\mathrm{hi}}\leftarrow 1$. Double $\nu_{\mathrm{hi}}$ until $p\left(\nu_{\mathrm{hi}}\right)\leq p_{\max}$, subject to $i_{b,max}$ trials. If no feasible upper bound is found, report failure and terminate the update.  9:       Bisect $\left[\nu_{\mathrm{lo}},\nu_{\mathrm{hi}}\right]$ until the feasible candidate meets (52) at the stated relative target. Set $\nu^{★}\leftarrow \nu_{\mathrm{hi}}$; report failure if the fixed budget is exhausted.10:    **end if**11:    Form the candidate ${\tilde{w}}_{n,t}\left(\nu^{★}\right)$.12:    Apply any required radial feasibility correction and verify the inner gap bound after that correction.13:    Evaluate (44).14:    **if** the candidate decreases the objective by more than $\epsilon_{\mathrm{mon}}$ **then**15:       Retain the preceding feasible iterate and terminate.16:    **else**17:       Accept the candidate and set i←i+1.18:    **end if**19:**until** $i\geq i_{\max}$ **or** $|f_{t}^{\left(i\right)}-f_{t}^{\left(i-1\right)}\left|/\max(1,\right|f_{t}^{\left(i-1\right)}\left|\right)\leq \epsilon_{\mathrm{fp}}$


### 3.6. Observed Channel State Information (CSI) and Action-Application Timeline

For imperfect observations, the controller receives a delayed estimate instead of the true $H_{t}$. It applies no prediction, temporal filtering, or robust compensation. For $0\leq \epsilon_{h}\leq 1$ and $∥H_{t}{∥}_{F}>0$, draw $Z_{t}$ independently of $H_{t}$, with independent CN(0,1) entries, and set $E_{t}=∥H_{t}{∥}_{F}Z_{t}/{∥Z_{t}∥}_{F}$. The observed matrix is

(56) $${\hat{H}}_{t}=\sqrt{1-\epsilon_{h}^{2}}H_{t}+\epsilon_{h}E_{t},\;\frac{E[∥{\hat{H}}_{t}-H_{t}{∥}_{F}^{2}|H_{t}]}{∥H_{t}{∥}_{F}^{2}}=2\left(1-\sqrt{1-\epsilon_{h}^{2}}\right).$$

Norm matching makes entries of $E_{t}$ dependent, so (56) describes a controlled perturbation rather than independent-entry additive estimation error. Its expected normalized mean-square error refers to observation time; later channel evolution adds mismatch before action application. The simulator uses the true channel norm to set the perturbation magnitude, without modeling a pilot estimator, quantizer, or feedback link.

For the longer-period study, let $T_{u}$ be the update period, $T_{f}$ the feedback delay, $T_{c}$ the modeled computation delay, and δ the service-integration step. At an update opportunity, an idle controller snapshots the current queue and the estimate timestamped $T_{f}$ earlier. If that observation is not yet available or the controller is busy, the opportunity is skipped. A new action becomes available after $T_{c}$, rounded up to the next integration boundary. Until then, the preceding feasible action remains active; before the first action, transmission is zero. An action that violates currently observable visibility is replaced by zero.

Real channels and queue service continue during feedback and computation. CSI age is measured from the observation underlying the active action and action age from its decision snapshot; both include the subsequent hold interval. In the long-slot study, arrivals and queue aging occur every 50 ms, while channels and service advance on a 0.25 ms grid. This finer step resolves the environment dynamics independently of the algorithm’s execution time. We compare ideal $T_{f}=T_{c}=0$ operation with a fixed feedback delay and computation delays calibrated from server measurements. The latter jointly change observation age and action-application phase, so the comparison tests the complete timing condition; zero-delay heuristic operation is not an optimal-service bound.

## 4. Reference Evaluation

This section defines the shared evaluation framework and presents controlled numerical comparisons under the parametric reference channel. In particular, the stage-specific comparisons distinguish the effects of introducing age information into cell selection and precoding under the prescribed channel coefficients. Section 5 evaluates performance under the normalized aperture model, with explicitly linked antenna gain, beamwidth, and spatial phase and a distinct experimental protocol.

### 4.1. Shared Setup and Performance Metrics

The shared geometry comprises one LEO satellite, 133 ground cells represented by one terminal each, and 133 cell-associated transmit ports. The 500 MHz downlink band is fully reused, with at most 19 concurrent streams. Section 2.3 defines the reference port channel; the physical model instead assigns these ports to the separate apertures described in Section 2.

The terrestrial footprint is centered at 1.18058° N, 21.77472° E. Version 4.5.0 of the H3 hierarchical geospatial indexing system [36] defines a reproducible discrete ground layout. A grid disk of radius two at resolution 2 yields 19 parent cells at this location, and the centers of their seven resolution-3 children define 133 fixed terminal locations. The 480 km orbit has a 53° inclination. Its sub-satellite point is aligned with the footprint center at the central reference slot of the 532-slot observation window. With the 20° minimum-elevation mask, all 133 cells are visible in every simulated slot. The visibility constraint is therefore nonbinding in these runs but remains part of both the model and implementation.

The reference channel follows Section 2.3; the shared traffic, queue, and service definitions are given in Section 2. Table 2 lists the principal parameters used in the parametric reference experiments unless a sensitivity variable is stated explicitly.

The default observation window comprises $t_{\mathrm{arr}}=512$ arrival slots (1.024 s) and $d_{\max}=20$ zero-arrival guard slots (0.040 s). Queues start empty. The guard gives every arrival its complete service and expiration horizon, and the terminal backlog must be below numerical tolerance. Goodput is normalized by the arrival duration, while mean power includes the full 1.064 s window.

An independently generated channel-and-traffic realization is the experimental unit. Within each realization, all methods share exogenous arrivals, geometry, and propagation, while each evolves its own queue under common resource constraints. Method comparisons first compute the metric difference within each realization, then construct uncertainty intervals from these differences across independent realizations. Fixed-state studies additionally hold the queue, channel, and selected set fixed where stated. The reference results use two-sided nominal 95% Student-*t* intervals, $\bar{x}\pm t_{0.975,N-1}s/\sqrt{N}$, where *x* denotes a metric or a within-realization difference and *N* counts independent realizations. Section 5.1 specifies simultaneous inference for the physical-model studies.

Let $t_{\mathrm{obs}}=t_{\mathrm{arr}}+d_{\max}$ denote the complete observation-window length. The primary reliability metric is the aggregate deadline-drop ratio

(57) $$\mathrm{dr}=\frac{\sum_{t=0}^{t_{\mathrm{obs}}-1}\sum_{n=0}^{n_{c}-1}l_{n,t}}{\sum_{t=0}^{t_{\mathrm{arr}}-1}\sum_{n=0}^{n_{c}-1}a_{n,t}}.$$

Timely goodput and average power are

(58) $$g_{\mathrm{timely}}=\frac{\sum_{t=0}^{t_{\mathrm{obs}}-1}\sum_{n=0}^{n_{c}-1}s_{n,t}}{t_{\mathrm{arr}}\Delta t},\;p_{\mathrm{avg}}=\frac{1}{t_{\mathrm{obs}}}\sum_{t=0}^{t_{\mathrm{obs}}-1}p_{t}.$$

Because the numerator of $g_{\mathrm{timely}}$ is measured in Mb and time is measured in seconds, $g_{\mathrm{timely}}$ is expressed in Mbps. Reported Gbps values differ only by the factor $10^{-3}$. For a fixed arrival realization with an empty terminal queue after the guard interval, timely goodput and deadline drop are complementary consequences of the same traffic-conservation identity. Goodput therefore supplies an operational throughput scale rather than an independent reliability test. Throughout the results, percent signs denote ratio levels or relative changes, whereas absolute differences between two drop ratios expressed as percentages are stated in percentage points. The energy per timely delivered bit is

(59) $$e_{b}=\frac{\Delta t\sum_{t=0}^{t_{\mathrm{obs}}-1}p_{t}}{10^{6}\sum_{t=0}^{t_{\mathrm{obs}}-1}\sum_{n=0}^{n_{c}-1}s_{n,t}}.$$

The aggregate drop ratio is defined for positive total arrival volume and energy per timely bit for positive total timely-service volume. Define the per-cell timely-delivery ratio as

(60) $$u_{n}=\frac{\sum_{t=0}^{t_{\mathrm{obs}}-1}s_{n,t}}{\sum_{t=0}^{t_{\mathrm{arr}}-1}a_{n,t}},\;J_{\mathrm{del}}=\frac{{\left(\sum_{n=0}^{n_{c}-1}u_{n}\right)}^{2}}{n_{c}\sum_{n=0}^{n_{c}-1}u_{n}^{2}}.$$

We use $u_{n}=0$ for cells with no arrivals and $J_{\mathrm{del}}=0$ when all delivery ratios are zero. For delay evaluation, timely service is resolved by queue-age stage. Traffic served from stage *d* has experienced delay (d+1)Δt. Within each realization, delay quantiles are weighted by timely served bit volume. Reported delay levels average these realization-specific quantiles; they are not quantiles pooled across all traffic. Delays are conditional on timely delivery and must be interpreted jointly with expiration loss. For warmed-up tests, arrival/drop/goodput statistics refer to the tagged measurement cohort, while RF energy and its timely-service denominator include all traffic served during measurement and guard. Warm-up itself is excluded. The fine-step experiment timestamps service on its integration grid rather than assigning every departure the end-of-slot delay.

### 4.2. Controllers and Reference Experimental Conditions

The baselines vary traffic awareness, deadline awareness, interference screening, and precoding while retaining the same channel, arrivals, and physical constraints:

1. **Round-Robin Equal (RR-Equal):** Visible nonempty cells are visited in cyclic index order, and the selected port-to-cell streams use equal-power diagonal transmission. This reference neither constructs a correlation graph nor inverts a channel matrix.
2. **Maximum-Queue RZF (MaxQueue-RZF):** At most $k_{\max}$ visible cells with the largest aggregate backlog $q_{n,t}$ are selected, followed by power-normalized RZF. It uses queue volume but ignores age and pairwise correlation.
3. **Earliest-Deadline-First RZF (EDF-RZF):** Cells are ordered first by their oldest nonempty age stage and then by backlog, followed by power-normalized RZF. It provides a deadline-aware scheduling reference without graph screening or iterative FP.
4. **Graph-Selected RZF (Graph-RZF):** Algorithm 2 is retained, while weighted FP is replaced by power-normalized RZF. The methods share the activation rule, while their selected sets evolve with their respective queues.
5. **Queue-Weighted Graph FP (Queue-Graph-FP):** The graph and FP stages are retained while replacing (38) by normalized aggregate backlog. This isolates the reciprocal-slack age term.

**Stage-specific urgency variants:** Age-Graph-Queue-FP uses (38) for graph selection and aggregate backlog for FP, whereas Queue-Graph-Age-FP makes the opposite substitution. Together with DAGFP and Queue-Graph-FP, these variants support an urgency-placement study that evaluates how age-resolved weighting affects the graph-selection and FP stages.

The five main baselines and DAGFP share bandwidth, power and stream constraints, causal observations, exogenous arrivals and channel states, queue accounting, and guard duration. Actual service is limited by the queued traffic even when the physical-layer rate is higher. The stage-specific variants are additional component controls.

The reference implementation uses unnormalized channels, absolute $10^{-12}$ safeguards for correlation and RZF, a $10^{-5}$ multiplier-search tolerance, and at most 50 FP iterations. The validation implementation adds noise normalization and the inner-solve accuracy bound.

The reference load sweep uses χ∈{1,20,28,32,36,40,60} with 20 independent realizations per load. The urgency-placement comparison uses the same 20 realizations at χ=36. Sensitivity cases use ten realizations per setting and cover $d_{\max}\in \left\{10,20,40\right\}$, β∈{0,0.5,1,2,4,8}, η∈{0.1,0.2,…,0.9}, v∈{0.1,1,10,30,100}, and $k_{\max}\in \left\{8,12,16,19,24\right\}$, together with the traffic and CSI conditions below. Reduced-state enumeration and runtime sampling are specified in Section 4.5.

Two nonstationary traffic profiles preserve the expected aggregate load of the stationary reference. Let $\bar{\lambda }=\left[\lambda_{\min}\left(\chi \right)+\lambda_{\max}\left(\chi \right)\right]/2$. In the moving-hotspot test, a group of $n_{h}=\mathrm{round}\left(0.2n_{c}\right)$ cells forms the active hotspot. Hotspot membership shifts cyclically every 128 slots. Background cells use $0.5\bar{\lambda }$, while

(61) $$\lambda_{h}=\frac{n_{c}\bar{\lambda }-\left(n_{c}-n_{h}\right)0.5\bar{\lambda }}{n_{h}}.$$

In the bursty test, every cell follows an independent two-state Markov-modulated Poisson process. Low-to-high and high-to-low transition probabilities of 0.02 and 0.08 give a stationary high-state probability of 0.2. The low and high rates are $0.25\bar{\lambda }$ and $4\bar{\lambda }$, which preserve $\bar{\lambda }$ in stationarity. The initial state is drawn from the stationary distribution.

CSI sensitivity uses delays $\tau_{\mathrm{csi}}\in \left\{0,1,2,5\right\}$ and the norm-matched perturbation model

(62) $${\hat{H}}_{t}=\sqrt{1-\epsilon_{h}^{2}}\;H_{t}+\epsilon_{h}E_{t},$$

where $\epsilon_{h}\in \left\{0,0.05,0.1,0.2\right\}$. Before rescaling, the entries of $E_{t}$ are independently drawn from CN(0,1); the realization is then normalized so that $∥E_{t}{∥}_{F}={∥H_{t}∥}_{F}$. Thus, $\epsilon_{h}$ is the perturbation coefficient rather than the realized relative Frobenius error $∥{\hat{H}}_{t}-H_{t}{∥}_{F}/{∥H_{t}∥}_{F}$. Cell selection and precoding use the delayed or perturbed estimate, whereas the achieved SINR is evaluated with the true channel.

### 4.3. Reference Performance and Stage Attribution

**Figure 2:**
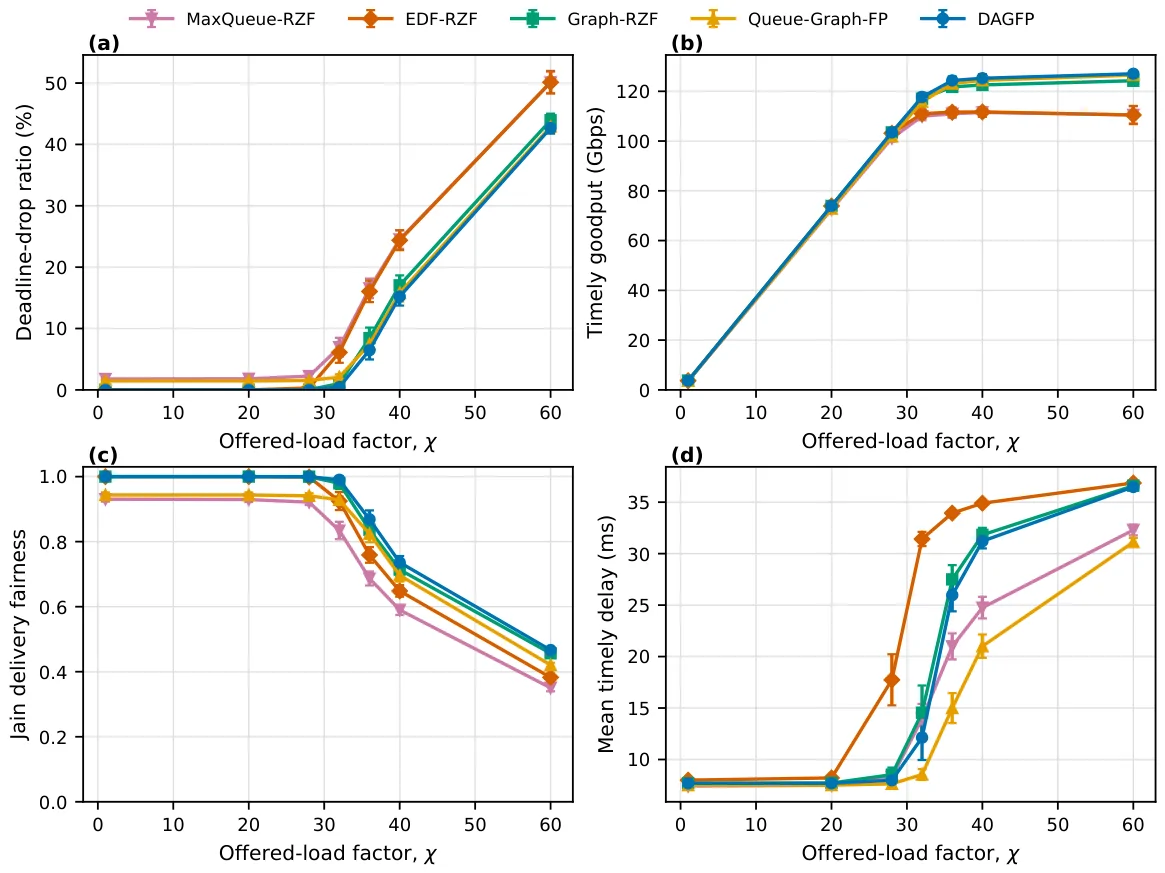
Parametric reference channel configuration. Load-dependent (**a**) deadline-drop ratio, (**b**) timely goodput, (**c**) delivery fairness, and (**d**) conditional timely-delivery delay. Markers show sample means, and error bars show two-sided 95% Student-*t* confidence half-widths over 20 independent channel-and-traffic realizations.

Figure 2a shows the load-dependent deadline-drop ratio. DAGFP remains essentially lossless through χ=28. Deadline losses emerge at χ=32 and increase thereafter, which identifies the transition from a service-rich regime to deadline-limited congestion. Table 3 compares all methods at the transition load χ=36, including RR-Equal, whose high drop ratio would compress the other curves in Figure 2.

At χ=36, DAGFP reduces mean deadline drop by 1.951 percentage points relative to Graph-RZF and by 0.958 percentage points relative to Queue-Graph-FP, with higher timely goodput and delivery fairness (Table 3). Graph-RZF shares DAGFP’s graph-selection rule but uses RZF instead of FP precoding.

The urgency-placement comparison in Table 4 identifies active-set construction as the main source of the observed age-weighting increment. Adding age information only to graph selection reduces mean drop by 0.980 percentage points. Changing only the FP weight shifts the observed mean by 0.044 and 0.022 percentage points under backlog-based and age-aware selection, respectively.

The reliability improvement involves power and delay trade-offs. Relative to Queue-Graph-FP, DAGFP uses 10.39 W more mean reference port power, while the mean realization-specific 95th-percentile timely-delivery delay increases from 26.9 to 35.7 ms.

At χ=60, DAGFP’s drop reduction relative to Queue-Graph-FP narrows to 0.226±0.182 percentage points. The smaller increment under heavy overload is consistent with fewer opportunities for queue priorities to avert expiration when service capacity is scarce. The stronger age-weighting effect in this reference sweep occurs near its congestion transition.

### 4.4. Traffic, CSI, and Parameter Sensitivity

**Figure 3:**
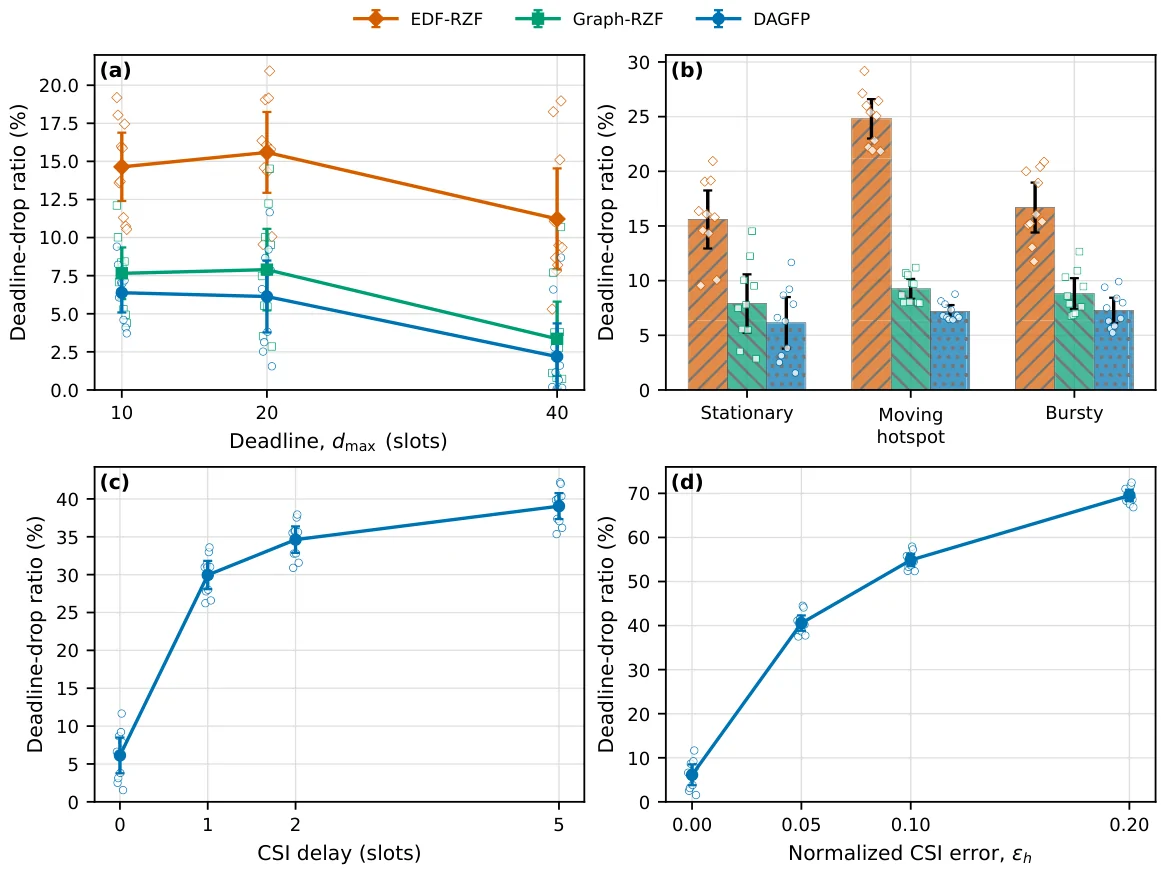
Parametric reference channel configuration. Deadline-drop ratio at χ=36 under variations in (**a**) traffic lifetime, (**b**) demand dynamics, (**c**) CSI delay, and (**d**) the norm-matched additive CSI perturbation coefficient. In panel (**b**), orange, green, and blue bars represent EDF-RZF, Graph-RZF, and DAGFP, respectively. Open markers show results from ten independent channel-and-traffic realizations per setting. Lines and bars show sample means, and error bars show two-sided 95% Student-*t* confidence half-widths.

Longer deadlines give the scheduler more opportunities to absorb demand fluctuations (Figure 3a). Increasing $d_{\max}$ from 10 to 40 reduces DAGFP’s mean drop from 6.382% to 2.190%; it remains below Graph-RZF and EDF-RZF at both endpoints. These reference-model comparisons are consistent with contributions from both the selection rule and the precoder over the tested lifetime range.

The moving-hotspot and bursty profiles preserve expected aggregate demand while changing its temporal distribution (Figure 3b). Both graph-based methods have lower observed loss than EDF-RZF, and DAGFP retains a lower sample mean than Graph-RZF. The ordering is consistent with value from compatibility-aware selection and an additional contribution from FP under these reference conditions.

**Figure 4:**
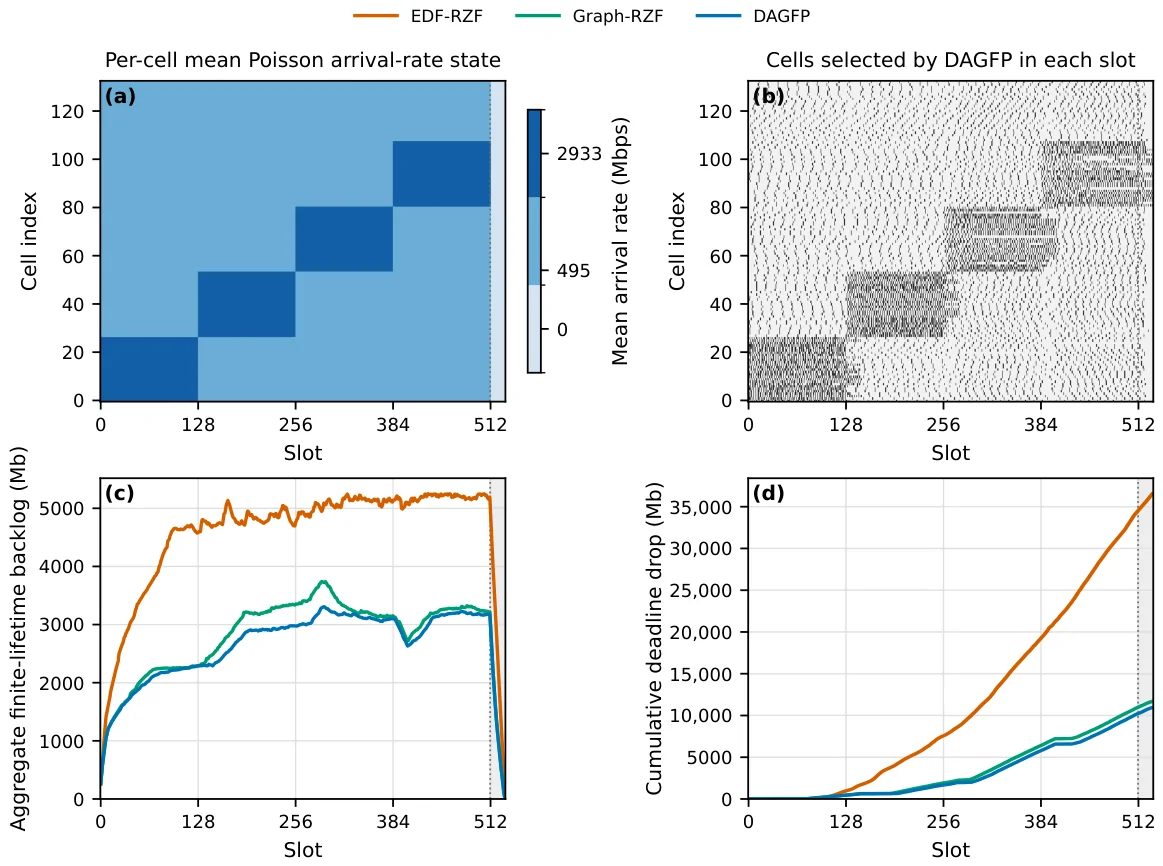
Parametric reference channel configuration. Moving-hotspot trajectory for one channel-and-traffic realization at χ=36: (**a**) per-cell mean Poisson arrival-rate state, (**b**) cells selected by DAGFP in each slot, (**c**) aggregate start-of-slot finite-lifetime backlog, and (**d**) cumulative deadline drop aligned with the corresponding expiration slot. The three colors in (**a**) represent the zero-arrival guard, background, and hotspot rate states. The dark pixels in (**b**) denote selected cells. The curves in (**c**,**d**) retain all 532 recorded slots without smoothing. Panel (**d**) uses a post-step representation. The dotted vertical line marks the start of the guard interval, and shading identifies this interval in (**c**,**d**).

Figure 4 shows selections shifting toward newly loaded blocks while residual traffic keeps earlier hotspots competitive. Channel opportunity and compatibility determine joint service; DAGFP and Graph-RZF share the selection rule but differ in precoding and subsequent queue evolution. During the guard, selections contract as queues drain, while unserved traffic can still expire.

CSI mismatch has a much larger effect in the reference configuration (Figure 3c,d): one-slot delay raises mean drop from 6.133% to 29.956%, and perturbation coefficient $\epsilon_{h}=0.2$ raises it to 69.539%. Both graph construction and FP use the complex channel directly, so mismatch can affect selection and interference suppression together. Section 5.5 examines all six methods under the physical model.

The reference parameter scans separate scheduling preferences from payload resources. The graph threshold trades admitted concurrency against channel correlation: the default η=0.2 yields lower observed drop than either the restrictive value 0.1 or the looser value 0.3. Introducing age weights improves delivery and fairness; retaining age information is more consequential in this reference setting than strongly amplifying it.

Increasing the power weight *v* lowers mean reference port power at the cost of higher drop, expressing a reliability–power preference within the same hard constraints. Increasing $k_{\max}$ instead expands the available concurrency: the 24-stream case improves delivery but assumes more streams than the default payload budget of 19. Section 5.3 examines these choices under the physical model.

### 4.5. Reference Selection Quality and Computation

**Figure 5:**
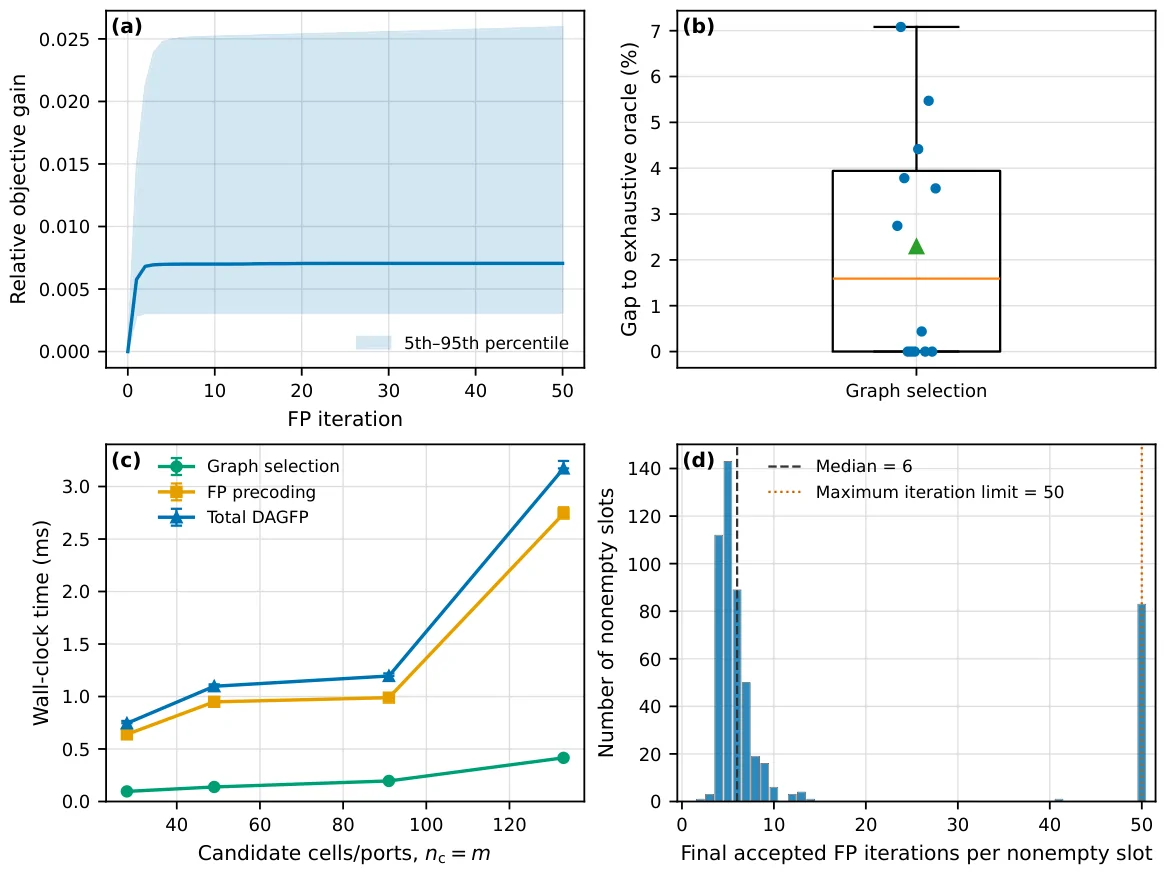
Parametric reference channel configuration. Numerical behavior of DAGFP: (**a**) median normalized FP objective improvement with a 5th–95th percentile band across nonempty slots, (**b**) reduced-state graph-selection gap over 12 channel states, (**c**) controller-core execution-time scaling over 50 repetitions of one fixed state at each $\left(n_{c},k_{\max}\right)$ pair, and (**d**) distribution of the final accepted FP iteration count over 531 nonempty slots. For visualization in (**a**), a trajectory that stops early retains its final objective value at later iteration indices. In (**c**), markers connected by solid guides show medians, and upper whiskers show 95th percentiles. In (**b**), blue points show individual states, the orange line is the median, and the green triangle is the sample mean. In (**d**), the median is 6, the maximum iteration limit is 50, and 83 slots (15.63%) reach that limit.

Figure 5b shows that, across 12 reduced ten-cell channel states with at most three active streams, graph selection has a mean objective gap of 2.29% and a maximum gap of 7.08% relative to exhaustive subset enumeration with the same FP routine. These values provide a local benchmark at χ=36 under the reference model. The same finite FP solver evaluates every candidate subset in the tested ten-cell states.

The accepted FP objectives are nondecreasing within tolerance, and all recorded actions satisfy power and stream constraints. Figure 5d shows that 15.63% of the 531 nonempty decisions reach the 50-iteration limit; the median stopping residual is $8.00\times 10^{-6}$. Section 5.7 evaluates larger iteration budgets and a projected-stationarity diagnostic under the validation implementation.

Figure 5c measures controller-core scaling under Python 3.12.13 on an Intel Core i9-14900KF central processing unit (CPU). Each $\left(n_{c},k_{\max}\right)\in \left\{\left(28,4\right),\left(49,7\right),\left(91,13\right),\left(133,19\right)\right\}$ uses one preconstructed state and 50 timing repetitions, covering urgency, selection, and precoding. A separate complete 133-cell diagnostic realization has median/p95 decision times of 3.318/21.584 ms, both exceeding the reference slot duration. The 2 ms reference slots provide a fine-grained, idealized setting for evaluating how deadline-aware scheduling responds to evolving channel conditions and age-resolved queue states, with each decision assumed to be available immediately. Section 5.7 relates the cost drivers and implementation speed-up to service at a longer causal update period.

## 5. Performance Evaluation

The normalized aperture studies evaluate performance, component roles and sensitivity with physically linked gain, beamwidth and spatial phase, using the separate-aperture channel unless stated otherwise. The controlled model transition holds implementation and parameters fixed across channels. The reference and aperture studies otherwise use different numerical safeguards, parameter settings, and evaluation samples.

### 5.1. Physical-Model Protocol and Parameter Selection

The physical-model studies use the geometry, traffic model, constraints, and performance metrics defined in Section 4. Defaults are empty queues, a 1.024 s arrival window, a 0.040 s guard, and a common 40 ms deadline (Table 5). Controller-independent random streams implement the shared-input design in Section 4.1. Simulations use a common numerical environment; server timing uses separate state cohorts.

Each independent realization serves as the statistical unit, with method differences evaluated using paired observations as described in Section 4.1. The sensitivity studies of controller parameters, deadlines, dynamic traffic, service models, and iteration budgets use 100 independent realizations per condition. Metric levels, non-drop contrasts, and timing use descriptive two-sided 95% Student-*t* intervals. Drop contrasts use 95% Bonferroni simultaneous intervals within defined comparison families: main and ablation (25 each), retuned RZF (15), parameter and traffic (231), CSI (93), service (190), window (60), and longer-period (16).

Baselines and urgency variants follow Section 4.2. All six main methods share observations and resource constraints. Queue-Graph-FP remains backlog-only in both stages, with no β dependence. Top-K-FP retains DAGFP’s urgency, singleton scores, and FP solver without pairwise screening. Main comparisons use method-specific graph and FP parameters selected as described below; component ablations use common settings.

Parameter selection jointly searches η∈{0.1,0.2,0.3}, β∈{0.5,1,2} and v∈{0.01,0.1,1} where applicable: all three for DAGFP, η,v for Queue-Graph-FP, and η for Graph-RZF with β=1. Parameter selection uses ten development realizations, separate from evaluation, with equal weighting of loads 2 and 4. Selection minimizes mean RF power among candidates within 0.2 percentage points of the lowest mean drop. Table 5 lists the selected DAGFP and Graph-RZF settings; Queue-Graph-FP uses (η,v)=(0.3,1).

The main studies use RZF $\rho =10^{-3}$ in noise-normalized coordinates. A separate study searches $\left\{10^{-5},10^{-4},10^{-3},10^{-2},10^{-1},1,10,100\right\}$ on ten development realizations across all five loads; all three RZF baselines select ρ=1. Their evaluation uses 100 held-out realizations per load, separate from the main and CSI cohorts. Component ablations instead fix (η,β,v)=(0.3,1,1) wherever applicable.

### 5.2. Model Transition and Main Performance

The controlled comparison uses the same validation solver and parameters for the parametric reference channel, the pattern-adjusted channel and the normalized aperture channel. At load 36, DAGFP’s mean drop rises from 7.047% to 88.618% across the full transition, with most of the congestion shift already present after pattern correction. Similar aggregate drops after the two corrections do not isolate phase effects on spatial structure or method performance. The main comparison uses common loads χ∈{1,2,4,8,36} to cover the shifted operating range.

**Figure 6:**
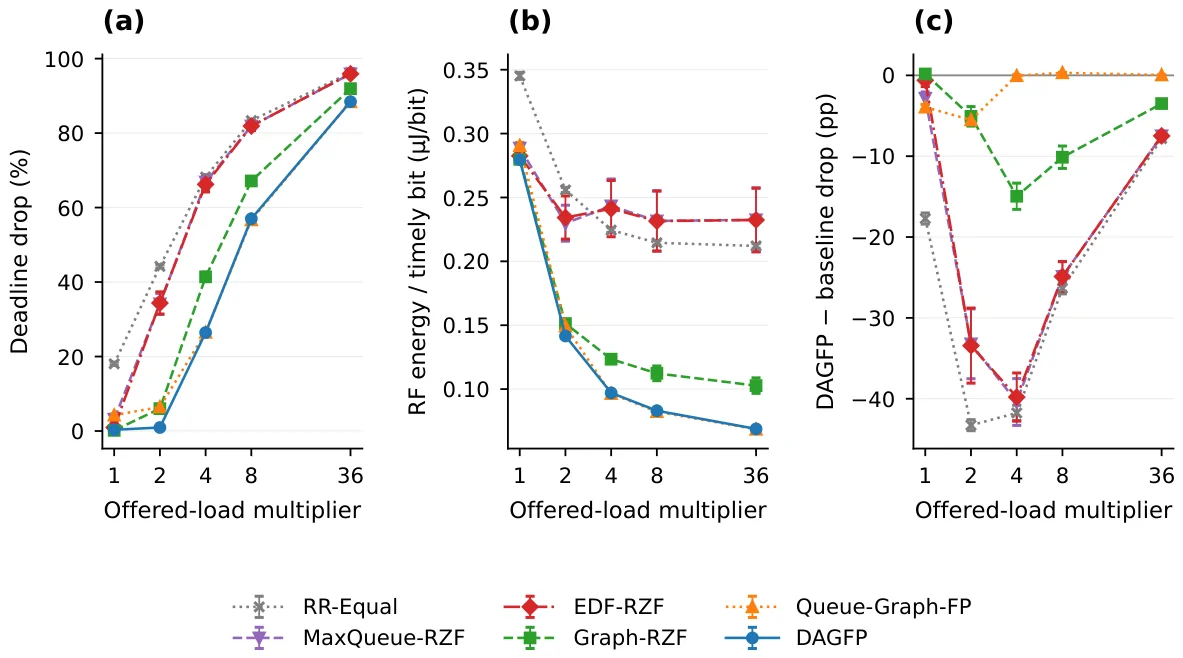
Six-method performance under the normalized aperture model, with common inputs across methods in each of 100 independent realizations per load and RZF $\rho =10^{-3}$: (**a**) deadline drop, (**b**) RF energy per timely bit, and (**c**) differences in drop. Metric-level intervals are descriptive 95% intervals; drop-difference intervals are 95% Bonferroni simultaneous intervals. Negative DAGFP-minus-baseline differences favor DAGFP.

Figure 6 tests the value of age weighting under the aperture model with the default deadline and ideal observation and computation. Starting from empty queues over a 1.024 s arrival window followed by a complete deadline guard, DAGFP reduces drop relative to Queue-Graph-FP by 5.511 percentage points at χ=2. Both retain graph selection and FP, so the contrast concerns age-resolved weighting. The difference at χ=4 is unresolved, while Queue-Graph-FP performs better at loads 8 and 36: age weighting’s benefit depends on service availability relative to demand.

Table 6 compares DAGFP with independently tuned RZF baselines using evaluation realizations separate from parameter selection. DAGFP reduces drop relative to Graph-RZF by 0.909 percentage points [0.765,1.053] at load 4 and outperforms all three retuned RZF methods at loads 4, 8, and 36. Retuned EDF-RZF is better at loads 1 and 2. The advantage over these stronger comparators is therefore narrower than in the fixed-coefficient study and concentrated at the evaluated congested loads.

### 5.3. Age Weighting, Selection and Precoding

**Figure 7:**
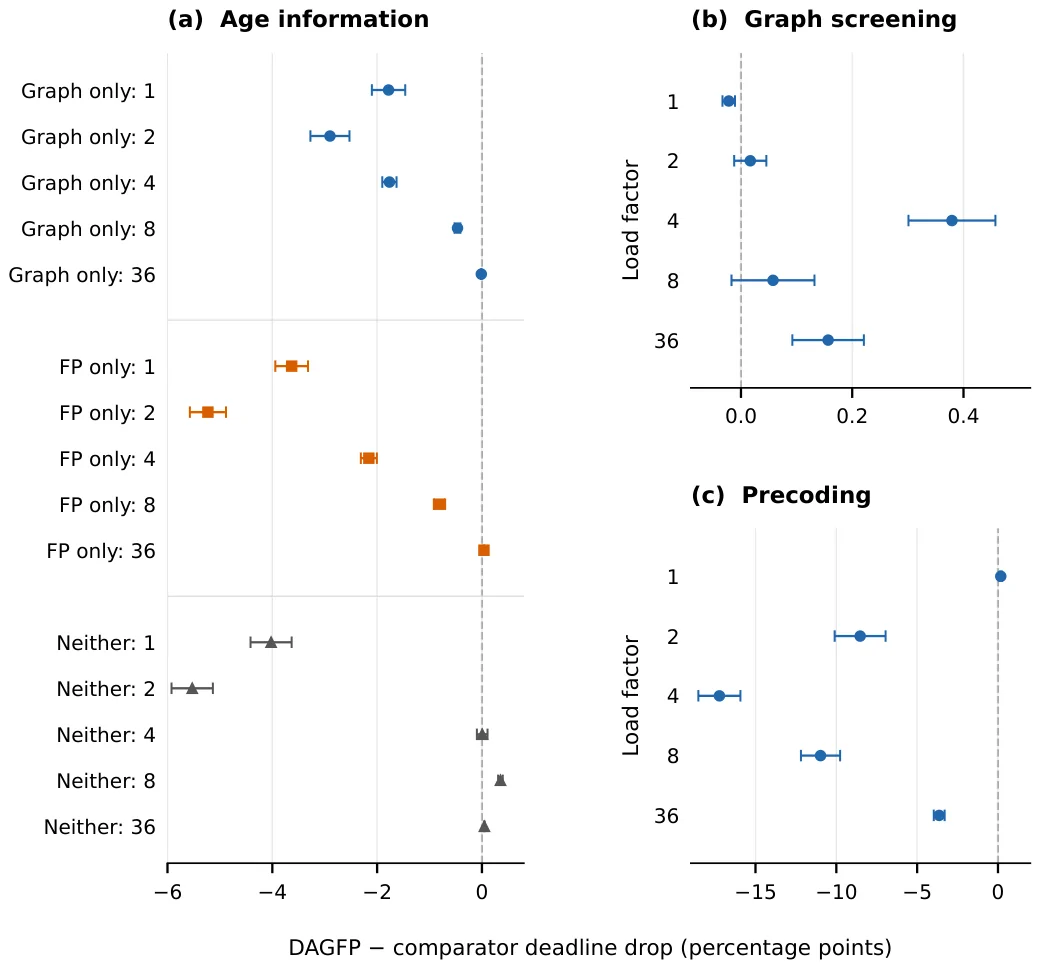
Common-parameter component comparisons: (**a**) age-information placement, (**b**) Top-K-FP without compatibility screening, and (**c**) Graph-RZF. In (**a**), “Graph only”, “FP only”, and “Neither” denote Age-Graph-Queue-FP, Queue-Graph-Age-FP, and Queue-Graph-FP, respectively. The numbers following these labels denote the load factor. Points show mean DAGFP-minus-comparator drop differences in percentage points; negative values favor DAGFP. Each comparison uses within-realization differences across 100 independent realizations, with 95% Bonferroni simultaneous intervals. Each method evolves its own queue and selected set during the simulation. Graph-RZF in panel (**c**) uses $\rho =10^{-3}$; Table 6 reports the separate comparison with independently tuned RZF baselines.

The common-parameter ablation tests how age placement interacts with precoding (Figure 7). At load 4, DAGFP reduces drop relative to age-in-graph-only and age-in-FP-only by 1.765 and 2.157 percentage points, respectively. The observed mean drop differs from backlog weighting in both stages by only 0.005 percentage points at load 4; age-in-FP-only performs better at load 36. Age contributions thus depend on their placement and operating condition, rather than adding independently across stages.

To isolate the precoding effect, queue, channel, urgency, and graph-selected set are fixed at three states from each of 100 independent physical-model DAGFP realizations at load 4. With common (η,β,v)=(0.3,1,1), $\rho =10^{-3}$ for both RZF and FP initialization, and at most 50 FP iterations, the dimensionless objective (39) increases by 10.686 [10.061,11.311] over RZF. This descriptive 95% interval uses within-realization averages of the three FP-minus-RZF differences. This increase measures the immediate improvement in the surrogate objective, while Figure 7 assesses service performance as queues evolve.

The graph comparison uses Top-K-FP to remove pairwise screening while retaining urgency scores and FP. DAGFP has lower drop at load 1, unresolved differences at 2 and 8, and higher drop at 4 and 36; its load-4 excess is 0.379 percentage points. Screening is therefore conditionally useful. Separately, eight-cell enumeration over all 93 subsets of at most three streams, including the empty set, measures subset quality with the same finite FP solver. Graph selection matches the best enumerated objective in most tested eight-cell states.

At load 4, the wider search grid η∈{0.1,0.2,…,0.9} yields a lower observed mean drop at η=0.8 than at the development-selected default η=0.3, which is used in the main comparisons. Increasing $k_{\max}$ from 8 through 12, 16, 19, and 24 improves service but changes the spatial resource budget, whose default remains 19.

### 5.4. Power, Energy, and Conditional Delay

At load 4, DAGFP uses 6.266 W more mean RF power than Queue-Graph-FP; RF energy per timely bit is 97.05 versus 96.48 nJ/bit. Its energy cost is lower at loads 1 and 2 but higher at 4, 8, and 36. These RF quantities exclude circuitry and power-amplifier direct-current (DC) consumption.

Increasing *v* above 1 at load 4 lowers RF output and raises drop for both FP controllers. Since equal penalties yield different mean power levels, the results characterize reliability–power trade-offs under the tested parameter settings. Supplementary Figure S1 presents the power–drop trade-offs over the tested power weights. At load 4, the mean realization-specific 95th-percentile timely-delivery delay is 39.84 ms for DAGFP and 36.64 ms for Queue-Graph-FP. These conditional delays are interpreted alongside deadline drop. Supplementary Table S1 reports RF power, energy per timely bit, and conditional delay across the main-study loads.

### 5.5. Deadline, Dynamic Traffic, and CSI Sensitivity

At load 4, the deadline study evaluates common deadlines of 20, 40, and 80 ms with β∈{0,0.5,1,2,4,8}. At the default β=1, DAGFP reduces drop by 1.853 percentage points relative to Queue-Graph-FP at the shortest deadline. Across the tested deadlines, β=0.5 yields the lowest observed mean drop.

The moving-hotspot and bursty traffic profiles vary demand distribution while preserving expected aggregate demand. At load 4, DAGFP reduces drop relative to Queue-Graph-FP by 2.545 and 0.999 percentage points under bursty and moving-hotspot traffic, respectively. These results demonstrate the benefit of age weighting under dynamic traffic conditions.

**Figure 8:**
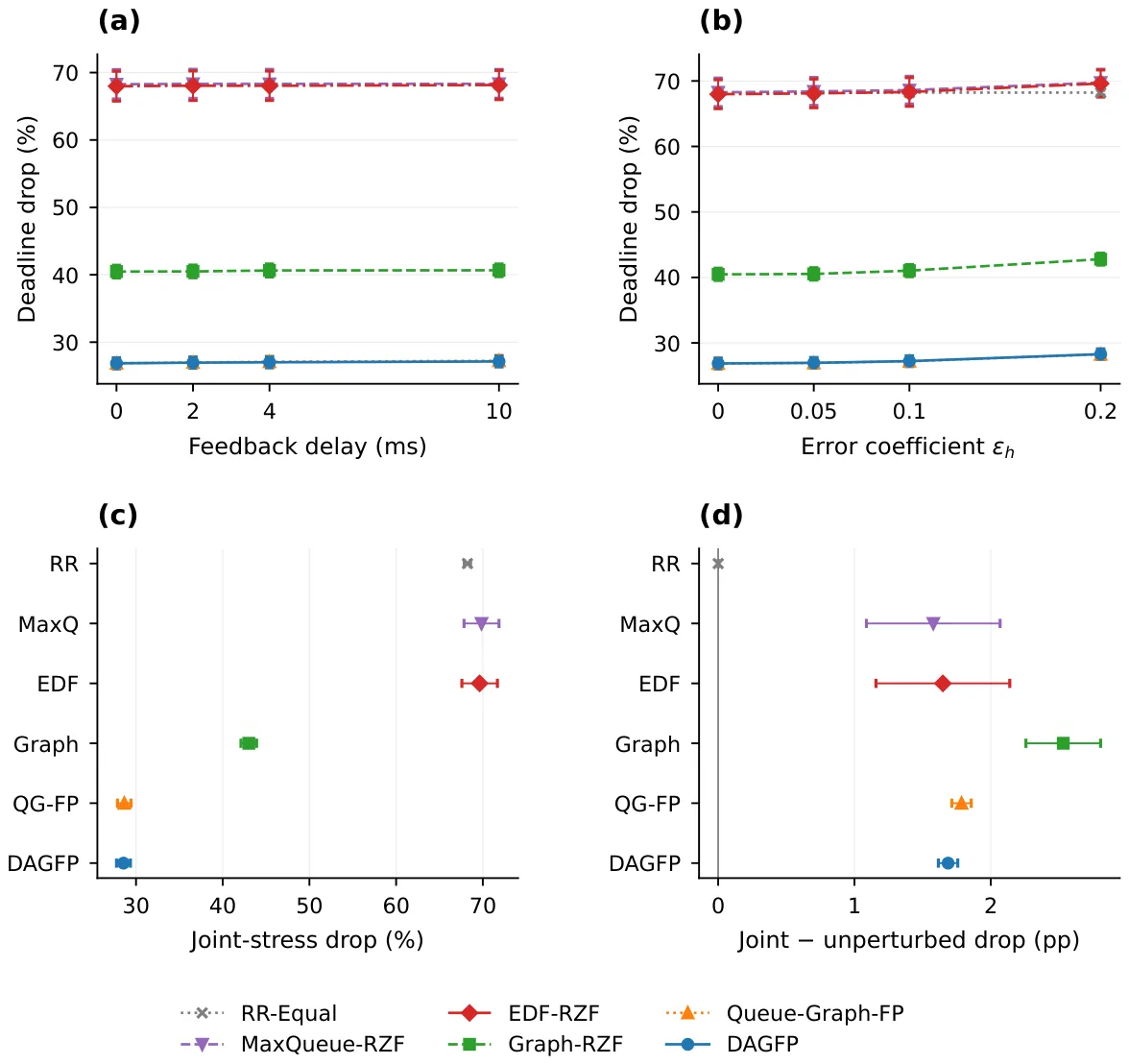
CSI sensitivity of six methods at load 4 with RZF $\rho =10^{-3}$: (**a**) feedback delay with $\epsilon_{h}=0$; (**b**) channel perturbation with zero feedback delay; (**c**) joint 10 ms feedback delay and $\epsilon_{h}=0.2$; and (**d**) each method’s joint-minus-unperturbed drop difference in percentage points. Each condition uses 100 independent realizations. Points show means. Intervals in (**a**–**c**) are descriptive 95% intervals; those in (**d**) are 95% Bonferroni simultaneous intervals from the 93-contrast CSI family. Positive differences in (**d**) indicate increased drop.

The slot-based CSI study evaluates all six methods under feedback delays up to 10 ms, perturbation coefficients up to 0.2 and their combined effects (Figure 8), with instantaneous computation. For a delay of *d* slots, the controller uses ${\hat{H}}_{\max\{0,t-d\}}$, reusing the first stored observation, including its perturbation, during startup. At load 4, relative to undelayed and unperturbed CSI, DAGFP’s mean drop increases by 0.275 percentage points with 10 ms delay alone and by 1.687 percentage points with the same delay and $\epsilon_{h}=0.2$.

In the normalized aperture model, each terminal’s common complex propagation factor multiplies all feed coefficients, leaving their relative structure determined by geometry. At fixed geometry and with a fixed precoder, this factor scales desired and interfering powers equally, but receiver noise is unchanged; SINR and scheduling decisions can therefore change. The observation perturbation in (56) is generally not a common terminal-wise scalar and can alter relative feed amplitudes and phases. Delay alone and joint delay–perturbation thus probe different forms of CSI mismatch in Figure 8. Over the evaluated arrival window, the normalized inner-product magnitude at 10 ms has a minimum of 0.999634 and a median of 0.999958 (Supplementary Table S2), indicating little change in feed-domain channel direction under the tested geometry, while common fading can still change received power.

### 5.6. Service Model and Observation Window

To examine the Shannon-rate assumption, actual queue service is computed as

(63) $$R_{n,t}^{\mathrm{svc}}=\zeta b_{\mathrm{sub}}\min\left\{\log_{2}\left(1+\gamma_{n,t}\right),r_{\max}\right\},\;r_{\max}=5.900855/1.1\approx 5.3644.$$

Here, $R_{n,t}^{\mathrm{svc}}$ is in bit/s, and the service volume in Mb is $10^{-6}R_{n,t}^{\mathrm{svc}}$ times the service duration in seconds. The rate ceiling follows the 256-ary amplitude and phase-shift keying (256APSK) 3/4 efficiency in European Telecommunications Standards Institute (ETSI) EN 302 307-2 V1.4.1 with roll-off 0.1 [8], while ζ∈{1,0.9,0.8} represents different overhead levels. The controller continues to optimize the Shannon-rate objective.

The rate ceiling alone leaves the evaluated performance unchanged. However, overhead increases congestion and changes the relative performance of the methods. At load 4, ζ=0.8 increases DAGFP’s mean drop from 26.866% to 38.111%, while Queue-Graph-FP achieves lower drop at both ζ=0.8 and ζ=0.9. Supplementary Figure S2 presents the service-model comparisons across the tested loads.

Window sensitivity is evaluated at load 4 with arrival durations of 1.024, 4.096, and 16.384 s, each followed by a complete deadline guard. Each configuration uses 30 independent realizations, starting from empty queues or after a 4.096 s warm-up. The drop difference is unresolved for the 1.024 s empty-start window, whereas Queue-Graph-FP achieves lower drop in both longer empty-start windows and in all warmed-up cases (Supplementary Table S3). These results indicate that observation duration and initial queue conditions influence the relative performance of the methods.

### 5.7. Numerical Reliability and Computational Feasibility

Spatial diagnostics distinguish the full selection channel from the selected precoding channel. At load 4, the normalized aperture model reduces the mean selected-channel condition number from 13.67 to 7.37 relative to the reference model. Noise normalization prevents numerical safeguards from suppressing aperture-model correlations. Equivalent channel and noise rescaling preserves selection and precoding decisions to numerical precision over the tested scales. Feasibility is maintained under the tested tolerance settings. Supplementary Tables S4 and S5 report the spatial diagnostics and numerical-threshold activation rates, respectively.

For the final accepted reduced precoder $W\in C^{k_{t}\times k_{t}}$, the FP auxiliary variables and matrices A,B are recomputed for (44). With $c=v/p_{\max}$, define G=B−AW−cW, $B=\{X:{∥X∥}_{F}^{2}\leq p_{\max}\}$, $\alpha ={(∥A∥}_{F}{+c)}^{-1}$, and $M=[W-\Pi_{B}\left(W+\alpha G\right)]/\alpha$. The relative projected-stationarity residual is

(64) $$\frac{{∥M∥}_{F}}{\max\{∥B{∥}_{F},∥AW+cW{∥}_{F},10^{-30}\}}.$$

This diagnostic evaluates stationarity for the selected precoder before action-format conversion.

The iteration-budget study examines 5–200 FP iterations at load 4 (Supplementary Figure S3). Budgets of 5 and 10 increase deadline drop relative to 50 iterations, with a 0.174-percentage-point increase at five iterations. At 50 iterations, the within-realization fraction of nonempty decisions reaching the iteration limit averages 4.24% across independent realizations, and the mean realization-level 95th percentile of the projected residual is $1.25\times 10^{-3}$.

Runtime is measured on an Intel Core i9-14900KF server using single-threaded libraries. Within each realization, all controllers are repeatedly timed on the same four fixed states from a Graph-RZF trajectory. Call-time quantiles are computed within each realization and then averaged over 100 independent realizations per load. Caching row energies alongside eigendecomposition reuse provides a mean speed-up of 1.20 for the reference solver: within each independent realization, the uncached median decision time is divided by the cached median decision time, and the resulting ratios are averaged. At load 4, reducing the validation controller’s iteration budget from 50 to 20 decreases the mean realization-level 95th-percentile decision time from 23.77 to 15.09 ms.

The longer-period experiment in Table 7 evaluates a 50 ms update period and a 200 ms common deadline, with 50 FP iterations, RZF $\rho =10^{-3}$, unperturbed CSI, and method-specific computation delays calibrated from server measurements. For each method, $T_{c}$ is the mean of the within-realization 95th-percentile decision times measured at load 4 under this fixed-state protocol. Feedback delay is 10 ms, and service advances on a 0.25 ms integration grid while the previous feasible action remains active during computation.

DAGFP reduces deadline drop relative to Queue-Graph-FP by 2.318 percentage points under ideal timing and 1.608 percentage points under fixed delays. Under fixed delays, it also outperforms Graph-RZF by 4.893 percentage points. These comparisons characterize service performance under the evaluated timing conditions. Introducing feedback and computation delays changes both observation age and action application timing, so the lower drop of the FP controllers cannot be attributed separately to either change.

## 6. Conclusions

This study addressed joint cell activation and precoding for dynamic multibeam LEO downlinks with spatially heterogeneous demand, finite traffic lifetimes, and co-channel interference. DAGFP uses a causal cross-layer framework in which deadline urgency derived from age-resolved service slack and instantaneous channel compatibility jointly shape cell activation. Weighted fractional programming then allocates power and suppresses residual interference on the selected subchannel under the corresponding traffic weights.

The controlled comparisons distinguish the effects of age weighting, channel-compatible selection, and precoding. Under the reference model, the incremental benefit of age-resolved weighting over backlog weighting is associated mainly with active-set construction. DAGFP also achieves a lower deadline-drop ratio than Graph-RZF, which shares the same graph-selection rule but employs RZF instead of FP precoding. Under the normalized aperture model, these effects depend on traffic and operating conditions for the modeled fixed-location terminals with single directional receive apertures and ideal satellite tracking. DAGFP outperforms independently tuned RZF baselines at the evaluated congested loads, whereas EDF-RZF performs better at lighter loads. Queue-Graph-FP achieves lower drop at loads 8 and 36 in the main study. In separate studies at load 4, it also achieves lower drop with payload fractions of 0.9 and 0.8, and with longer empty-start windows or warmed-up queues. Simpler Top-K selection also performs better in some evaluated conditions.

Delayed or perturbed CSI can degrade service performance because both selection and precoding rely directly on observed channel information. The iteration-budget and timing studies characterize computational cost and service performance, including operation with a 50 ms update period, a fixed feedback delay, and computation delays calibrated from server measurements.

Future work will integrate predictive and uncertainty-aware channel models, extend the formulation to multi-satellite and multiband coordination, and investigate implementation optimization.

## Figures and Tables

**Table 1:** Related formulations compared by traffic representation, decision variables, interference and channel information, and solution method.

Study	Traffic and Deadlines	Scheduling and Continuous Variables	Interference and CSI	Solution Method
Chen et al. [23]	Window-average demand	Illumination; complex precoding within selected clusters	Interference after precoding; instantaneous CSI and error tests	Prescribed regularized MMSE precoder; SDP, majorization or greedy illumination
Chen et al. [24]	Window-average demand	Illumination; scalar beam powers	Co-channel power interference; channel matrix over the scheduling window	Mean-field allocation; rounding and quadratic scheduling
Zhao et al. [28]	Finite traffic lifetime; age-indexed buffers	Multi-satellite hopping; scalar beam powers	Channel-gain observations; interference-aware power allocation	A3C hopping and MADDPG power allocation
Guo et al. [16]	Traffic classes; expiry and overflow	Association, hopping and subbands; scalar powers	Interference-graph frequency allocation	Annealing, ILP, graph allocation and water filling
DAGFP	Common deadline; age-resolved queues	Cell/feed selection; complex precoder matrix	Channel-correlation screening; delayed or perturbed CSI	Urgency-weighted graph selection and weighted FP

Abbreviations: CSI, channel state information; MMSE, minimum mean-square error; SDP, semidefinite programming; A3C, asynchronous advantage actor–critic; MADDPG, multi-agent deep deterministic policy gradient; ILP, integer linear programming; DAGFP, deadline-aware graph-guided fractional precoding; FP, fractional programming.

**Table 2:** Parametric reference channel configuration. Principal simulation parameters.

Parameter	Value	Parameter	Value
LEO altitude $h_{\mathrm{leo}}$	480 km	Carrier frequency $f_{c}$	12 GHz
Orbit inclination	53°	Minimum service elevation	20°
Allocated downlink bandwidth $b_{\mathrm{sub}}$	500 MHz	Slot duration Δt	2 ms
Number of service cells $n_{c}$	133	H3 parent/child resolution	2/3
Cell-associated transmit ports *m*	133	H3 grid-disk radius/children per parent	2/7
Concurrent-stream limit $k_{\max}$	19	Reference port-power budget $p_{\max}$	1000 W
Reference transmit/receive power-gain scale	40/40 dB	Effective noise power spectral density	−204 dBW/Hz
Nominal transmit/receive angular width	16°/16°	Transmit/receive response suppression floor	20/25 dB
Zenith rain mean/std.; slant cap	1/1/20 dB	Shadow-fading standard deviation	2 dB
Rain/shadow correlation time	2/0.5 s	Diffuse-component correlation time	5 ms
Rician *K* at 0°/90°	25/30 dB	Traffic lifetime $d_{\max}$	20 slots (40 ms)
Per-cell mean-rate bounds at χ=1	[5,50] Mbps	Graph threshold η	0.2
Age coefficient β	1	Normalized power weight *v*	0.1
FP stopping tolerance/iteration limit	$10^{-5}/50$	Multiplier tolerance/iteration limit	$10^{-5}/80$
Objective acceptance tolerance	$10^{-8}$	Traffic-generation/guard slots	512/20
Realizations per load-sweep/sensitivity case	20/10		

**Table 3:** Parametric reference channel configuration, evaluated with the reference implementation without receiver-noise normalization. Performance at the congestion-transition load χ=36. Values are means ± two-sided 95% confidence half-widths over 20 realizations.

Method	Drop (%, ↓)	Goodput (Gbps, ↑)	Fairness (↑)	Timely Delay (ms)
RR-Equal	94.395±0.076	7.453±0.002	0.486±0.032	38.95±0.08
MaxQueue-RZF	16.544±1.597	110.974±1.694	0.687±0.021	20.99±1.26
EDF-RZF	16.048±1.746	111.628±1.858	0.759±0.024	33.94±0.27
Graph-RZF	8.441±1.715	121.763±2.005	0.837±0.028	27.50±1.38
Queue-Graph-FP	7.448±1.480	123.080±1.584	0.824±0.026	15.00±1.45
DAGFP	6.490±1.535	124.351±1.621	0.869±0.027	25.98±1.58

**Table 4:** Parametric reference channel configuration, evaluated with the reference implementation without receiver-noise normalization. Urgency-placement analysis at χ=36. “Age” denotes the reciprocal-slack urgency in (38), whereas “backlog” denotes normalized aggregate backlog. Values are the means ± two-sided 95% confidence half-widths over 20 independent realizations.

Graph Weight	FP Weight	Drop (%, ↓)	Goodput (Gbps, ↑)	Fairness (↑)	Timely Delay (ms)
Backlog	Backlog	7.448±1.480	123.080±1.584	0.824±0.026	15.00±1.45
Backlog	Age	7.492±1.495	123.022±1.609	0.824±0.026	15.04±1.46
Age	Backlog	6.468±1.547	124.379±1.597	0.869±0.028	25.93±1.63
Age	Age (DAGFP)	6.490±1.535	124.351±1.621	0.869±0.027	25.98±1.58

**Table 5:** Normalized aperture validation configuration. Sensitivity variables and the separate 50 ms operating point are stated with their studies. Both terminal and feed patterns use the normalized aperture model.

Parameter	Value	Parameter	Value
Altitude/inclination	480 km/53°	Carrier/bandwidth	12 GHz/500 MHz
Cells/feeds	133/133	Stream limit	19
Minimum elevation	20°	Aggregate feed RF power	1000 W
Full HPBW	16°	Radiation efficiency	0.6
Aperture diameter	0.09236 m	Peak gain	19.053 dBi
Feed-center pitch	Twice the diameter	Receiver pointing	Ideal terminal tracking
Noise density	−204 dBW/Hz	Reference slot/deadline	2 ms/40 ms
Zenith rain mean/std.; slant cap	1/1; 20 dB	Shadow standard deviation	2 dB
Rain/shadow time constants	2/0.5 s	Diffuse time constant	5 ms
*K* factor	$\left(25+5e_{n,t}/90{}^{\circ}\right)$ dB	Arrival/guard slots	512/20
Mean-rate bounds at χ=1	5–50 Mbps/cell	Loads χ	1, 2, 4, 8, 36
DAGFP (η,β,v)	(0.3,1,1)	Graph-RZF η	0.2
FP relative stopping tolerance	$10^{-5}$	FP iteration budget	50
Relative inner gap tolerance	$10^{-10}$	Dual search budget	80
Objective acceptance tolerance	$10^{-8}$	Main realizations/condition	100

**Table 6:** Physical-model deadline-drop ratios (%) with independently tuned RZF baselines. Each load uses 100 independent realizations shared across methods and separate from parameter tuning. All three RZF baselines use ρ=1, while DAGFP retains its main-study settings. The ideal-observation evaluation samples differ from those in Figure 6; method comparisons use 95% Bonferroni simultaneous intervals.

Load	DAGFP	EDF-RZF	Graph-RZF	MaxQueue-RZF
1	0.280	0.069	0.631	2.141
2	0.862	0.221	1.842	3.799
4	26.419	36.204	27.328	36.496
8	57.011	66.331	57.894	66.441
36	88.413	91.513	89.172	91.509

**Table 7:** Mean deadline-drop ratio (%) at χ=4 with a 50 ms update period and 200 ms deadline. Each condition uses 30 independent realizations and a 10 s arrival window. Fixed timing includes 10 ms feedback delay and the listed computation delay $T_{c}$; ideal timing assumes zero delays. The RZF methods use $\rho =10^{-3}$. Table 6 reports independently tuned RZF with ρ=1 under different timing conditions; performance relative to these tuned baselines under the present timing conditions is therefore not assessed.

Method	$T_{c}$ (ms)	Ideal Timing	Fixed Delays
RR-Equal	0.825	72.103	71.869
MaxQueue-RZF	0.887	69.426	69.510
EDF-RZF	0.915	69.293	69.520
Graph-RZF	0.904	43.217	43.201
Queue-Graph-FP	24.893	45.796	39.916
DAGFP	23.766	43.478	38.308

## Data Availability

No external dataset was used in this study. The simulation results supporting the reported findings are presented in the article and its Supplementary Material. Further information may be obtained from the corresponding author upon reasonable request.

## Supplementary Materials

The following supporting information can be downloaded at: https://www.mdpi.com/article/10.3390/s26216733/s1, Figure S1: Reliability–RF-power sensitivity of DAGFP and Queue-Graph-FP under the normalized aperture model at load 4, with 100 independent realizations per controller and powerweight; Figure S2: Service-model sensitivity under the normalized aperture model; Figure S3: Finite-budget service and stopping under the normalized aperture model at load 4, with 100 independent realizations per condition; Table S1: RF power, energy and timely-delivery delay under the normalized aperture model, using 100 independent realizations per load in the main study; Table S2: Temporal direction similarity of the full 133-feed true channel vectors under the normalized aperture configuration used for the CSI study; Table S3: Window and initial-state sensitivity under the normalized aperture model at load 4; Table S4: Spatial diagnostics at load 4. Values are means ± between-realization standard deviations after averaging four captured states within each of 100 independent realizations; Table S5: Floor activation at load 4, averaged over four captured states within each of 100 independent realizations.
