# Supplementary material for: Deadline-Aware Graph-Guided Beam Hopping and Precoding for Dynamic Low Earth Orbit Downlinks
Source: Sensors (Basel). 2026 Oct 24;26(21):6733. doi: 10.3390/s26216733 (PMC13765938; doi:10.3390/s26216733)
Supplement: Supplementary file 1 [file sensors-26-06733-s001.zip › sensors-4580873-supplementary.pdf]

Supplementary Material

Deadline-Aware Graph-Guided Beam Hopping and Precoding for Dynamic Low Earth Orbit Downlinks

Chenyu Wu, Yuhang Li, Baoji Wang, Guocheng Lv and Ye Jin

Reliability and RF power

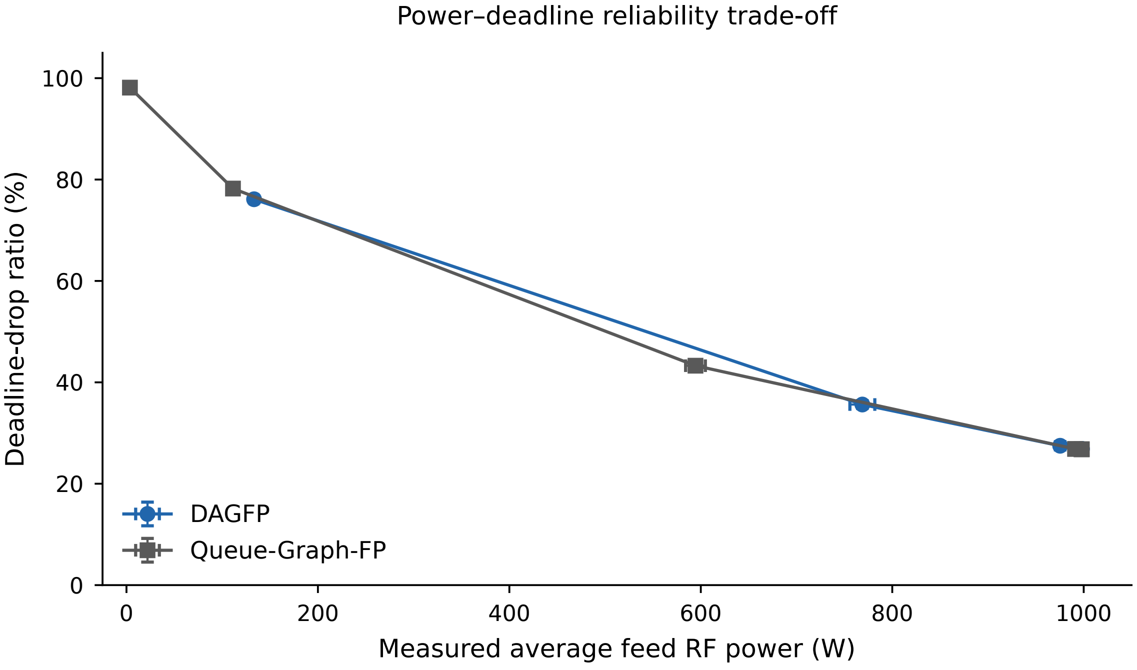

Figure S1: Reliability–RF-power sensitivity of DAGFP and Queue-Graph-FP under the normalized aperture model at load 4, with 100 independent realizations per controller and power weight. Both series use $\nu \in \{0.01, 0.1, 1, 10, 30, 100\}$; some low-weight points nearly coincide. Horizontal and vertical error bars show marginal descriptive 95% intervals.

Service-model sensitivity

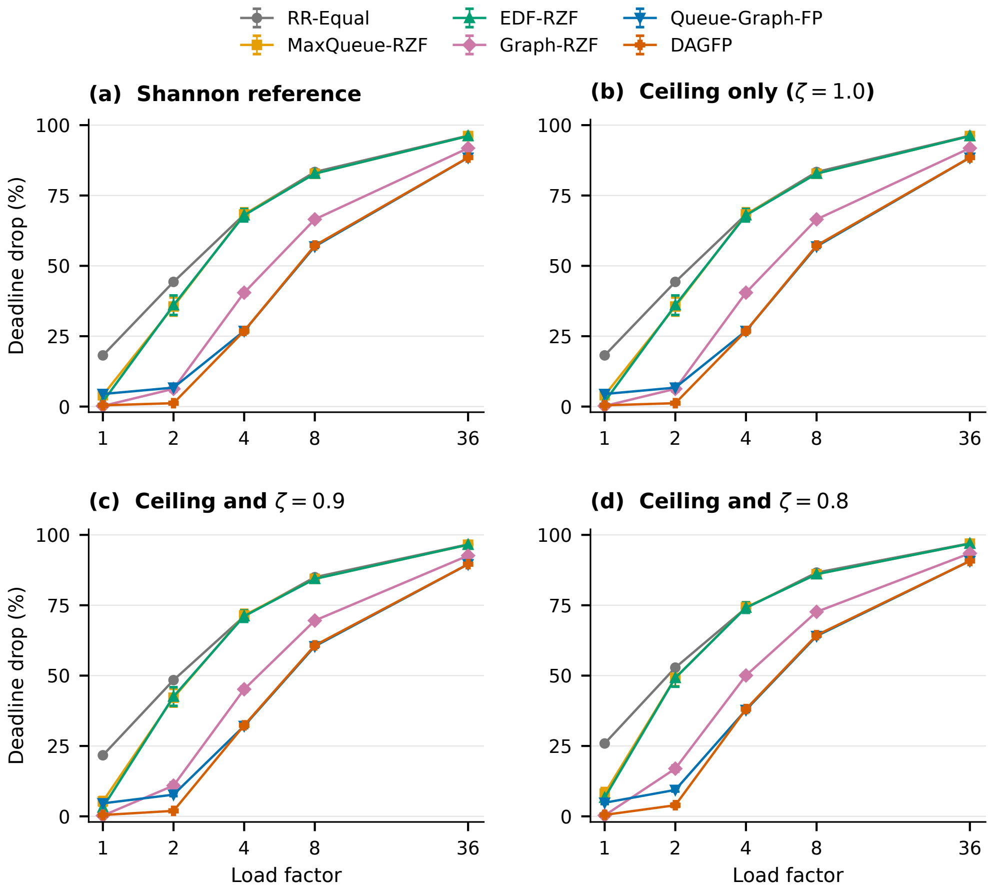

Figure S2: Service-model sensitivity under the normalized aperture model. All six methods are evaluated with uncapped Shannon service, the spectral-efficiency ceiling $r_{\max} = 5.900855/1.1 \approx 5.3644$ bit/s/Hz without additional payload loss, and the same ceiling with payload fractions $\zeta = 0.9$ and 0.8. The controllers continue to optimize the Shannon-rate objective. Each point averages 100 independent realizations, with descriptive 95% intervals.

At load 4, the DAGFP-minus-Queue-Graph-FP drop differences are 0.2137 and 0.3166 percentage points at $\zeta = 0.9$ and 0.8, respectively, with 95% Bonferroni simultaneous intervals $[0.1274, 0.3000]$ and $[0.2413, 0.3920]$ from the 190-contrast service-model family.

Finite iteration budgets

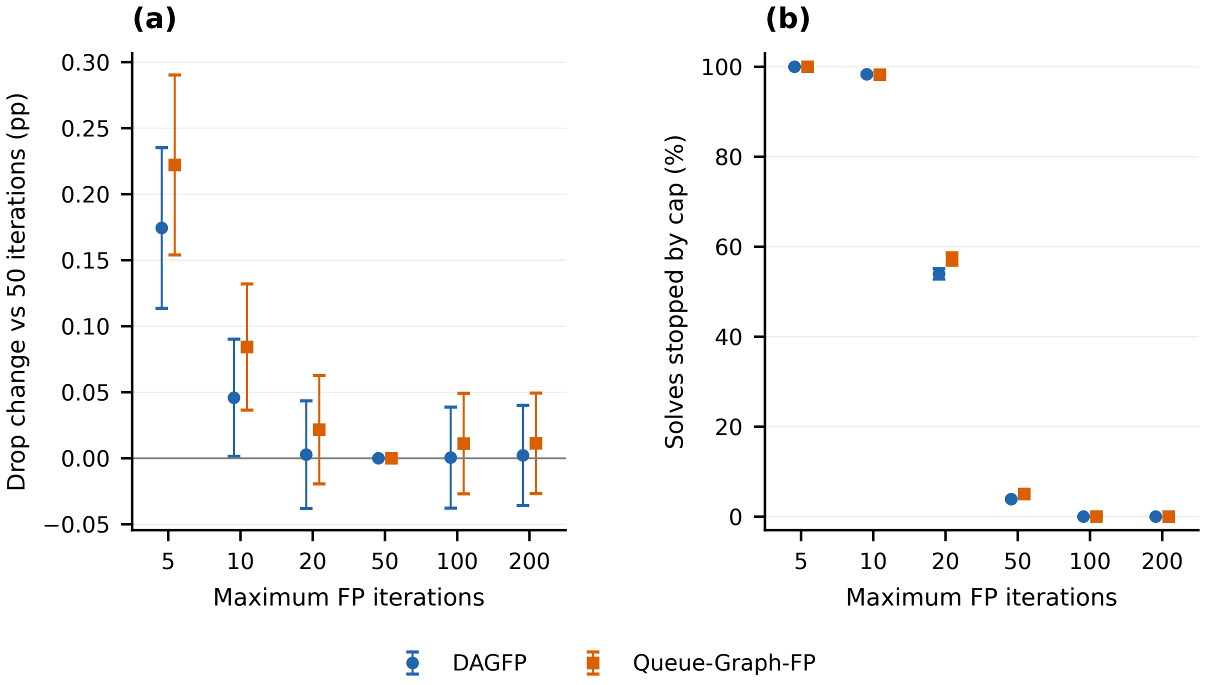

Figure S3: Finite-budget service and stopping under the normalized aperture model at load 4, with 100 independent realizations per condition. (a) Drop differences relative to the same method at budget 50, in percentage points, with 95% Bonferroni simultaneous intervals; positive values indicate increased drop. (b) The fraction of nonempty decisions stopped because of the iteration cap, calculated within each realization and then averaged, with descriptive 95% intervals. Decisions satisfying the stopping tolerance on the final allowed iteration are excluded from the cap-triggered fraction.

Power, energy and conditional delay

Load	Method	RF power (W)	Energy (nJ/bit)	p95 delay (ms)	p99 delay (ms)
1	DAGFP	975.055	279.6201	22.380	30.720
1	Queue-Graph-FP	973.035	290.5579	22.780	32.620
2	DAGFP	981.199	141.5533	27.680	34.560
2	Queue-Graph-FP	976.603	149.2327	26.840	35.360
4	DAGFP	997.198	97.0531	39.840	40.000
4	Queue-Graph-FP	990.932	96.4818	36.640	39.960
8	DAGFP	997.199	83.1131	40.000	40.000
8	Queue-Graph-FP	995.424	82.3440	40.000	40.000
36	DAGFP	997.204	68.8536	40.000	40.000
36	Queue-Graph-FP	996.279	68.5237	40.000	40.000

Table S1: RF power, energy and timely-delivery delay under the normalized aperture model, using 100 independent realizations per load in the main study. Entries are means of realization-level RF power, RF energy per timely bit, and bit-volume-weighted timely-delay quantiles. The RF quantities exclude circuitry and power-amplifier DC consumption. Delay quantiles are conditional on successful delivery and are averaged across realizations rather than pooled across traffic.

Temporal channel direction

For each terminal, we compute the magnitude of the normalized inner product between its full 133-feed true channel vectors at slots t and $t - d$:

$$c_n(t, d) = \frac{|\mathbf{h}_n(t)^{\mathrm{H}}\mathbf{h}_n(t - d)|}{\|\mathbf{h}_n(t)\|_2 \, \|\mathbf{h}_n(t - d)\|_2}. \tag{S1}$$

The common nonzero propagation factors cancel, while the signed feed-pattern amplitudes and geometric phases are retained. We evaluate $d = 0, 1, 2, 5$ over the 512-slot arrival window, using $t \geq d$ and nonzero norms. The formal CSI configuration has one shared geometry trajectory, counted once; the summaries describe terminal–time combinations and are not confidence intervals. Startup observation reuse, guard slots and observation perturbations are excluded.

Delay (ms)	Valid combinations	No full history	Zero norm
0	68096	0	0
2	67963	133	0
4	67830	266	0
10	67431	665	0

Delay (ms)	Minimum	5th percentile	Median	95th percentile	Maximum
0	1.000000000	1.000000000	1.000000000	1.000000000	1.000000000
2	0.999751724	0.999995328	0.999998330	0.999999878	0.999999964
4	0.999745283	0.999981328	0.999993330	0.999999506	0.999999846
10	0.999633531	0.999883059	0.999958253	0.999996919	0.999999048

Table S2: Temporal direction similarity of the full 133-feed true channel vectors under the normalized aperture configuration used for the CSI study. Statistics summarize valid terminal–time combinations within the 512-slot arrival window, using only times with a complete history of the specified delay and nonzero channel norms. Startup observation reuse and observation perturbations are excluded. Repeated copies of an identical geometry trajectory are not counted as additional samples. Zero-delay values equal one to within 7×10^{-16} numerical error.

Observation window and initialization

Arrival slots	Warm-up slots	DAGFP drop (%)	Difference [interval]
512	0	26.782	−0.022 [−0.266, 0.222]
512	2048	28.041	0.518 [0.290, 0.747]
2048	0	28.294	0.223 [0.105, 0.341]
2048	2048	28.618	0.352 [0.237, 0.468]
8192	0	28.636	0.290 [0.207, 0.374]
8192	2048	28.722	0.331 [0.243, 0.419]

Table S3: Window and initial-state sensitivity under the normalized aperture model at load 4. Differences are DAGFP minus Queue-Graph-FP in percentage points, with 95% Bonferroni simultaneous intervals from the 60-contrast family. Each configuration uses 30 independent realizations. Each arrival window is followed by a complete deadline guard; warm-up traffic is accounted for separately. The 512, 2048 and 8192 arrival slots correspond to 1.024, 4.096 and 16.384 s, respectively; 2048 warm-up slots correspond to 4.096 s.

Spatial structure

The pattern-adjusted configuration changes the antenna patterns while retaining the reference phase structure. The shared-fading configuration applies a common terminal-specific fading factor without the geometric feed phase. The complete aperture configuration includes both shared fading and geometric feed phase.

For each realization, four states are captured from a DAGFP trajectory generated with the reference implementation under the parametric reference channel. The channel configurations are compared using common queue states and exogenous inputs, while noise-normalized DAGFP selects the active set separately for each configuration using the settings in Table S4.

Channel	Correlation	Effective rank	Full condition	Selected condition
Original channel	0.05440 ± 0.00034	73.411 ± 2.314	811.905 ± 1235.645	13.670 ± 7.346
Pattern corrected	0.02534 ± 0.00020	74.369 ± 2.515	688.709 ± 1516.616	7.990 ± 3.040
Shared fading, no feed phase	0.08721 ± 1.2×10^{-17}	24.747 ± 0.881	155641.567 ± 49419.086	4.821 ± 0.819
Complete aperture model	0.02305 ± 4.0×10^{-18}	74.755 ± 2.470	761.336 ± 203.266	7.368 ± 2.914

Table S4: Spatial diagnostics at load 4. Values are means ± between-realization standard deviations after averaging four captured states within each of 100 independent realizations. Correlation is the full-channel median over distinct terminal-row pairs. Effective rank is the exponential entropy of normalized squared singular values. Condition numbers are ratios of the largest to the smallest singular value of the full and selected channel matrices, respectively. Full matrices are 133 × 133; selected matrices are $k_t \times k_t$, $k_t \leq 19$. The same noise-normalized DAGFP rule, $(\eta, \beta, \nu) = (0.3, 1, 1)$, selects for each model.

Numerical thresholds

Channel	Matrix	Raw scale (%)		Normalized (%)	
		Correlation	RZF	Correlation	RZF
Original	Full	0	0	0	0
Original	Selected	0	34.5	0	0
Complete	Full	99.9992	100	0	0
Complete	Selected	100	100	0	0

Table S5: Floor activation at load 4, averaged over four captured states within each of 100 independent realizations. Correlation columns give the percentage of distinct row pairs at or below the 10^{-12} norm-product threshold. RZF columns give the percentage of matrices activating the 10^{-12} regularization floor. Noise-normalized DAGFP chooses the selected set, held fixed between raw and normalized probes. Original denotes the reference channel; Complete denotes the normalized aperture channel. The trace-based RZF coefficient is $\rho = 10^{-3}$ in both coordinate systems.
